# Human Pancreatic Carcinoma-Associated Fibroblasts Promote Expression of Co-inhibitory Markers on CD4^+^ and CD8^+^ T-Cells

**DOI:** 10.3389/fimmu.2019.00847

**Published:** 2019-04-24

**Authors:** Laia Gorchs, Carlos Fernández Moro, Peter Bankhead, Katharina P. Kern, Imrul Sadeak, Qingda Meng, Elena Rangelova, Helen Kaipe

**Affiliations:** ^1^Department of Laboratory Medicine, Karolinska Institutet, Stockholm, Sweden; ^2^Department of Pathology/Cytology, Karolinska University Hospital, Stockholm, Sweden; ^3^Division of Pathology, Centre for Genomic & Experimental Medicine, Western General Hospital, Institute of Genetics & Molecular Medicine, University of Edinburgh, Edinburgh, United Kingdom; ^4^Department of CLINTEC, Karolinska Institutet, Stockholm, Sweden; ^5^Pancreatic Surgery Unit, Center for Digestive Diseases, Karolinska University Hospital, Stockholm, Sweden; ^6^Clinical Immunology and Transfusion Medicine, Karolinska University Hospital, Stockholm, Sweden

**Keywords:** cancer-associated pancreatic stellate cells, fibroblasts, pancreatic cancer, T-cells, co-inhibitory markers, PD-1, TIM-3

## Abstract

Carcinoma-associated pancreatic fibroblasts (CAFs) are the major type of cells in the stroma of pancreatic ductal adenocarcinomas and besides their pathological release of extracellular matrix proteins, they are also perceived as key contributors to immune evasion. Despite the known relevance of tumor infiltrating lymphocytes in cancers, the interactions between T-cells and CAFs remain largely unexplored. Here, we found that CAFs isolated from tumors of pancreatic cancer patients undergoing surgical resection (*n* = 15) expressed higher levels of the PD-1 ligands PD-L1 and PD-L2 compared to primary skin fibroblasts from healthy donors. CAFs strongly inhibited T-cell proliferation in a contact-independent fashion. Blocking the activity of prostaglandin E_2_ (PGE_2_) by indomethacin partially restored the proliferative capacity of both CD4^+^ and CD8^+^ T-cells. After stimulation, the proportion of proliferating T-cells expressing HLA-DR and the proportion of memory T-cells were decreased when CAFs were present compared to T-cells proliferating in the absence of CAFs. Interestingly, CAFs promoted the expression of TIM-3, PD-1, CTLA-4 and LAG-3 in proliferating T-cells. Immunohistochemistry stainings further showed that T-cells residing within the desmoplastic stromal compartment express PD-1, indicating a role for CAFs on co-inhibitory marker expression also *in vivo*. We further found that PGE_2_ promoted the expression of PD-1 and TIM-3 on T-cells. Functional assays showed that proliferating T-cells expressing immune checkpoints produced less IFN-γ, TNF-α, and CD107a after restimulation when CAFs had been present. Thus, this indicates that CAFs induce expression of immune checkpoints on CD4^+^ and CD8^+^ T-cells, which contribute to a diminished immune function.

## Introduction

The late diagnosis and the lack of an effective treatment brings pancreatic cancer to the fourth leading cause of cancer-related death, with a 5-year survival rate of only 6% (1). During the last decade, immunotherapy drugs have offered significant benefit for certain malignancies including melanoma, lung cancer, and head and neck cancer, but it has so far been unsuccessful for pancreatic cancer patients (2, 3). The failure of developing a successful treatment is due in part to the limited understanding of the complex microenvironment in the pancreatic tumor.

The most common form of pancreatic cancer, pancreatic ductal adenocarcinoma (PDAC), is characterized by a voluminous desmoplastic reaction mediated by carcinoma associated pancreatic stellate cells and other cancer-associated fibroblasts (CAFs). Under normal conditions, pancreatic stellate cells have a limited proliferative capacity and store vitamin A containing lipid droplets in their cytoplasm. In the presence of cancer cells, pancreatic stellate cells acquire an increased contractile ability which promotes the expression of α-smooth muscle actin (α-SMA), podoplanin and the loss of their characteristic cytoplasmic lipid droplets which results in the pathological release of extracellular matrix proteins triggering fibrosis and building a “wall” for therapy delivery (4). Therefore, the presence of large amounts of CAFs are associated with poor prognosis (5). Recent studies also suggest that there are several subtypes of CAFs in pancreatic cancer that may play different roles in the tumor microenvironment (6–8).

The importance of CAFs in tumors has mainly been studied in the context of interactions with tumor cells (9, 10). CAFs express the catabolic enzyme indoleamine 2,3-dioxygenase (IDO) and release a variety of factors such as transforming growth factor- β (TGF- β), vascular endothelial growth factor (VEGF), interleukins (IL)-6, IL-1 and IL-8, and prostaglandin E_2_ (PGE_2_) that directly or indirectly participate in the development of an immunosuppressive tumor milieu that promote tumor growth, angiogenesis and metastasis (10). However, a growing number of studies perceive fibroblasts as key contributors of immune evasion and show the direct interplay between fibroblasts and immune cells. Cancer associated fibroblasts (CAFs) induce T-regulatory cells as well as T-cell apoptosis in head and neck squamous cell carcinoma (11), modulate natural killer cell functions in melanoma (12), educate dendritic cells into regulatory cells in hepatic carcinoma (13) and induce protumoral M2 macrophages in PDAC (14). However, the interactions between CAFs and T-cells in pancreatic cancer remain largely unexplored.

The presence of both CD4^+^ and CD8^+^ tumor infiltrating T lymphocytes (TILs) is a predictor of long term survival in PDAC (15, 16). However, in the majority of cases cancer cells readily evade immune surveillance with the help of immune checkpoints. The general notion is that the chronic stimulation by tumor antigens and the exposure of suppressive cytokines, drive TILs to differentiate into an exhausted phenotype characterized by the expression of immune checkpoint molecules such as programmed cell death-1 (PD-1), T-cell immunoglobulin and mucin-domain containing-3 (TIM-3), lymphocyte-activation gene- 3 (LAG-3), cytotoxic T-lymphocyte-associated antigen-4 (CTLA-4), and T-cell Ig and ITIM domain (TIGIT). These exhausted T-cells have a diminished proliferative ability and lose their cytotoxic functions (17). Supporting this evidence a recent study shows that high levels of CD8^+^ PD-1^+^ TILs is correlated with poor prognosis and shorter overall survival in PDAC (18).

The fact that the T-cells can be held back by the tumor stroma in pancreatic cancer (19, 20) suggests that CAF-derived factors may be able to modulate T-cell functions and phenotype. Here, through a series of *in vitro* experiments we demonstrated that CAFs induce expression of immune-checkpoints on CD4^+^ and CD8^+^ T-cells, which contribute to a diminished immune function.

## Material and Methods

### Patients and Samples

Pancreatic tumor tissues were collected from 15 patients undergoing surgery at the Pancreatic Surgery Unit at Karolinska University Hospital, Huddinge, Sweden (Table 1). Thirteen of the patients had PDAC, one had adenosquamous carcinoma of the pancreas and one had colloid carcinoma of the pancreas. Primary normal skin fibroblasts were obtained from healthy donors and peripheral blood samples were collected from healthy blood donors. Written informed consent was obtained from the patients. The study was approved by the regional review board of ethics in research of Karolinska Institutet (entry nos. 2009/418-31/4, 2013/977-31.3, and 2017/722-32).

**Table 1:** Patient characteristics.

**Variables**	***n* = 15**
**DEMOGRAPHIC CHARACTERISTICS**	
**Age, years**	
Female gender, *n*	8
Median age, years (range)	64 (55–82)
Male gender, *n*	7
Median age, years (range)	52 (34–78)
**ONCOLOGIC CHARACTERISTICS**	
**Histological type**	
Pancreatic ductal adenocarcinoma, *n*	13
Adenosquamous carcinoma, *n*	1
Colloid carcinoma of the pancreas, *n*	1
**Tumor size, cm**	
>2 and ≤5	11
>5	4
**Tumor depth**, ***n***	
T3	13
T4	2
**Lymph node metastasis**, ***n***	
N0	1
N1	14
**Metastasis**, ***n***	
M0	12
M1	3
**Chemotherapy (FOLFOX)**, ***n***	
Yes	3
No	12

*T (3,4), Tumor stage; N1, lymph node with cancer; M1, Distant metastasis*.

### Cell Isolation

For isolation of pancreatic CAFs, a similar outgrowth method as first described by Bachem et al. was used (21). Freshly resected pancreatic tumor tissues were cut into small pieces and cultured in Dulbecco's modified Eagle's medium (DMEM) (GE Healthcare, cat no. SH3002101) supplemented with 10% fetal bovine serum (HyClone, cat no. SV3016003) and 1% penicillin-streptomycin (HyClone, cat no. SV30010) (complete DMEM) in 6 well plates. Fibroblasts were let to migrate out the tissue fragments for 15–20 days and then cells were harvested with trypsin-EDTA (HyClone, cat no. SH3023602) and transferred to T75 or T175 flasks for further expansion. Skin-derived fibroblasts were isolated from punch biopsies in the same fashion. Cells were expanded until passage 3 and then cryopreserved in complete DMEM with 10% dimethyl sulfoxide (GmbH cat no. WAK-DMSO-70) until analysis. Before the experiments, cells were thawed in complete DMEM medium and characterized by flow cytometer using the antibodies shown in Supplementary Table S1. Peripheral blood mononuclear cells (PBMCs) were isolated from buffy coats from healthy donors and patients by density gradient over Lymphoprep^TM^ gradient (Axis Shield, cat no. 1114547).

### Proliferation Assays

PBMCs were labeled with carboxyfluorescein succinimidyl ester (CFSE) (Molecular Probes, Life Technologies, cat no. C34554) (5 μg/ml) in PBS at 37°C for 15 min. After washing, the cells were plated in 96-well plates (2 × 10^5^), 24-well plates (1 × 10^6^), or 12-well plates (2 × 10^6^) (Corning) in the presence or absence of 30Gy irradiated CAFs at a 1:10 ratio (1 CAF per 10 PBMCs). The culture medium used was RPMI-1640 (HyClone, cat no. SH3025501) supplemented with 10% human AB serum and 1% penicillin-streptomycin. PBMCs were stimulated with OKT3 (25 ng/ml) (Biolegend, cat no. 317315) or left unstimulated. To prevent contact between fibroblasts and PBMCs, a 12-well Transwell system [0.4 μm pore size membrane (Corning)] was used for a set of experiments. Fibroblasts were added to the upper chamber and PBMCs were added to the lower chamber. On day 5, PBMCs were harvested and analyzed by flow cytometry. The majority of the proliferation assays were performed with allogeneic PBMCs, but a number for experiments were done with PBMCs and CAFs derived from the same patient.

Blocking experiments were performed in 96-well plates and 20 μM indomethacin [(Sigma-Aldrich, cat no. I7378) for PGE_2_ blocking], 1 mM 1-methyl-DL-tryptophan [(Sigma Aldrich, cat no. 860646) for IDO blocking], 10 μg/ml anti-PD-L1 and 10 μg/ml anti-PD-L2 (BioLegend cat no. 329710 and 345504), 5 μg/ml anti-TGF-β (R&D systems, cat no. MAB246) or the appropriate isotypes [IgG1/IgG2b (R&D systems, cat no. MAB002 and MAB004)] or vehicles were added to the cell cultures. To control for the effect that these substances may have on the proliferative response of T-cells in the absence of CAFs, a ratio of the response of T-cells in the presence and absence of CAFs was calculated for the control and the blocking conditions, respectively.

To study the interactions between CAFs, tumor cells, and PBMCs, we used the pancreatic tumor cell line PANC-1. The experiments were performed as described above, including irradiation of PANC-1 cells and CAFs at 30Gy, but the CAFs or PANC-1 cells were added at a ratio of 1 cell per 20 PBMCs (1:20).

For intracellular detection of cytokines, 25 ng/ml PMA, 1 μg/ml ionomycin, 10 μg/ml Brefeldin-A (Sigma-Aldrich, cat no. 79346, I0634, and B7651), and Golgi Stop (diluted 1:1,500, BD Biosciences, cat no. 554715) were added. PGE_2_ (0.1 μg/ml, Sigma Aldrich, cat no. P0409) was added to PBMC cultures for a set of experiments.

### Flow Cytometry

A list for the antibodies used in this study is shown in Supplementary Table S1. Extracellular staining was performed in CliniMACS PBS/EDTA buffer (Miltenyi Biotech cat no. 200-070-025) supplemented with 0.1% bovine serum albumin. Extracellular markers were analyzed on viable cells (7-AAD negative). Intracellular staining of α-SMA, IFN-γ, and TNF-α was performed using the BD Cytofix/Cytoperm^TM^ kit (BD Biosciences, cat no. 554714) according to the manufacturer's instructions. Intracellular staining for FOXP3 was done with FOXP3 staining buffer set (eBioscience, cat no. 0052300) according to the manufacturer's instructions. Phycoerythrin (PE) conjugated CD107a was added in the cell cultures together with PMA/I, Brefeldin A, and GolgiStop on day 5 for 6 h. Boolean gating strategy was used to study the frequency of CD4^+^ and CD8^+^ T-cells that were triple positive for TIM-3, PD-1 and CTLA-4 or for TIM-3, PD-1, and LAG-3. A FACSCanto (BD) was used for data acquisition and FlowJo (Tree Star, Ashland, OR, USA) version 10.2 was used for data analysis. Sub-gating for all analyzed samples was done with more than 100 events, and no data points were excluded due to low events.

### Immunohistochemistry Stainings

Fresh tumor tissue was sampled by specialized pancreatic pathologists immediately after surgical resection. The tissue sample was divided in two pieces: one was put in culture medium and sent for cell isolation, while the other was fixed in 4% formalin, embedded in paraffin and processed for histology and immunohistochemistry as matched reference tissue. Four μm thick sections were immunohistochemically stained using a Leica BOND III automated immunostainer. The antibody panel consisted of CD3 (clone LN10, manufacturer Novocastra, product code NCL-L-CD3-565, dilution 1:100), α-SMA (1A4, Dako, M0851, 1:500) and PD-1 (NAT105, Cell Marque, 315M-96, 1:100). CD3 and α-SMA were combined by duplex immunohistochemistry and stained using DAB (brown) respective AP (red) chromogens to better visualize the spatial relation between T-cells and CAFs.

Quantitative analysis of CD3^+^ and PD-1^+^ cells was performed on the whole slide images using QuPath (22). Briefly, four rectangular regions of equal size containing tumor cells and stroma, but not benign ducts or acinar tissue, were defined on each CD3 and α-SMA stained slide. The tumor regions were annotated manually and validated by a specialized pancreatic pathologist (CFM). The rectangular regions for quantification and the tumor annotations within the regions were transferred to, corrected where needed, and validated in the matched PD-1 stained slides. Afterwards, the several tumor annotations were combined and a stroma annotation was derived for each quantification region. Tissue areas and cells were detected and quantified for each region. The cells positive for CD3 or PD-1, respectively, according to DAB staining, were classified as residing within the tumor nests or within the stroma according to the annotation regions. Finally, the positive cells close to tumor cells were identified using Delaunay triangulation with a distance of 20 μm. Quantitative data was exported in a tabular format for descriptive statistical analysis. An overview of the image analysis pipeline is provided in Supplementary Figure S7.

### ELISA

Concentrations of IL-10 were measured in the supernatants obtained after co-cultures with an in-house ELISA, using an anti-human IL-10 coating antibody (R&D, cat no. MAB2172), a secondary biotinylated goat anti-human IL-10 antibody (R&D, cat no. BAF217), a standard of recombinant human IL-10 (R&D, cat no. 1064-IL), and horseradish peroxidase (Sanquin, cat no. M2032) for the enzymatic reaction.

### Statistical Analysis

To detect differences across two groups, Wilcoxon matched-pairs signed rank test was used. Data are expressed as the median. To study differences between more than two groups, a Friedman's test followed by Dunn's test was used. Correlations between proliferation and expression of markers was performed by Spearman correlation test. A *p*-value of <0.05 was considered statistically significant. All statistics were done using GraphPad Prism version 7 (La Jolla CA, USA).

## Results

### Characterization of Cancer Associated Pancreatic Fibroblasts

**Figure 1:**
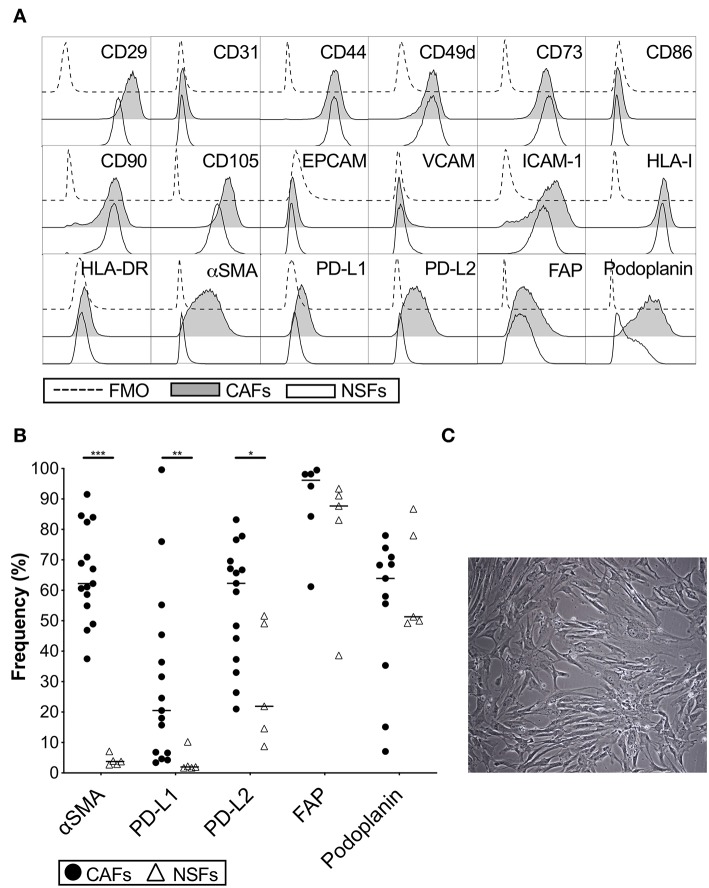
Phenotypic analysis of carcinoma associated pancreatic stellate cells (CAFs) and normal skin fibroblasts (NSFs) by flow cytometry. **(A)** Representative histograms showing different CAFs (gray) and NSFs (white) molecules expression compared to FMO controls (dashed line). **(B)** Comparison of α-SMA, PD-L1, PD-L2, FAP and podoplanin expression between CAFs (black dots) (*n* = 8–15) and NSFs (open triangles) (*n* = 5). **(C)** Representative image showing the morphology of CAFs at passage 3 (Original magnification × 10). All fibroblasts were characterized in passage 3. The bars indicate the median. Wilcoxon matched-pairs signed rank test was used to detect statistically significant differences *P < 0.05, **P < 0.01, ***P < 0.001.

The phenotype of primary CAFs was characterized by flow cytometry and compared to normal skin fibroblasts. Both types of fibroblasts were positive for the common stromal markers CD29, CD44, CD73, CD90, and CD105, but negative for the endothelial marker CD31 and the epithelial marker EPCAM (Figure 1A). CAFs and skin fibroblasts were both negative for VCAM, CD86 and HLA-DR, but positive for ICAM-1 and HLA class I (Figure 1A). However, only CAFs were positive for α-SMA (*p* < 0.0001) with a median expression of 62% (Figures 1A,B). The expression of both PD-L1 (*p* = 0.001) and PD-L2 (*p* = 0.01) was also higher in CAFs compared to skin fibroblasts (Figures 1A,B). We also noted that the expression of PD-L2 was generally higher compared to PD-L1 in both CAFs and normal skin fibroblasts. There was no statistically significant difference in the expression levels of fibroblast activation protein (FAP) and podoplanin (Figures 1A,B), which are markers known to be associated with cancer. To examine if the phenotype of CAFs is altered during serial passaging, the phenotype of CAFs from 3 to 6 donors were compared between passage 1, 2 and 3. No consistent difference was observed for the expression of α-SMA, PD-L1, PD-L2, or podoplanin at different passages (Supplementary Figure S1). The morphology of the isolated CAFs can be seen in a representative microphotograph in Figure 1C.

### Proliferative Capacity and Functionality of T-Cells Are Compromised in the Presence of CAFs

**Figure 2:**
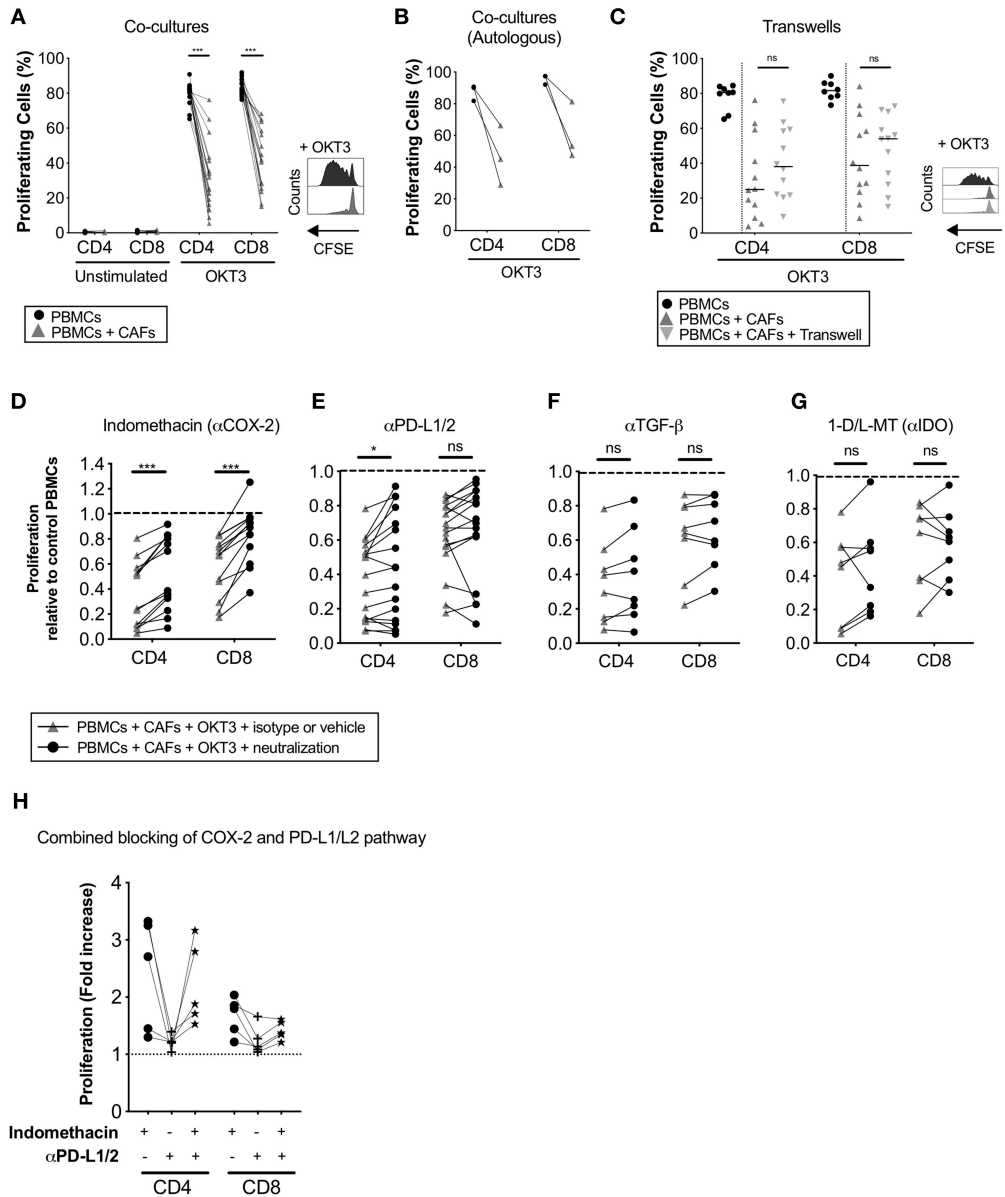
CAFs inhibit T-cell proliferation, but COX-2 inhibition partially restores T-cells proliferation. CFSE-labeled PBMCs were co-cultured in the absence (•) or presence (▴) of CAFs and stimulated with OKT3 (25 ng/ml) for 5 days. **(A)** Frequency of proliferating CD4^+^ and CD8^+^ T-cells in the absence or presence of allogeneic CAFs in unstimulated (*n* = 14) and stimulated (*n* = 18) conditions (left). Representative CFSE histograms on CD4^+^ T-cells (right). **(B)** Frequency of proliferating patient-derived CD4^+^ and CD8^+^ T-cells in the absence or presence of autologous CAFs (*n* = 3). **(C)** Frequency of proliferating CD4^+^ and CD8^+^ T-cells in direct co-cultures (▴), indirect transwell cultures (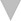) or without allogeneic CAFs (•) (*n* = 12) (left). Representative CFSE histograms on CD4^+^ T-cells (right). **(C–F)** Proliferating CD4^+^ and CD8^+^ T-cells was measured by flow cytometry after addition of corresponding isotype control or vehicle (▴) or the following blocking substances (•); **(C)** indomethacin to inhibit PGE_2_ production (*n* = 13), **(D)** anti-PD-L1 and anti-PD-L2 antibodies (*n* = 17), **(E)** anti-TGF-β antibodies (*n* = 8), or **(F)** 1-methyl-DL-tryptophan (1-D/L-MT) to inhibit indoleamine 2,3-dioxygenase (IDO) activity (*n* = 8). **(H)** COX2 inhibition, PD-L1 and PD-L2 blockade or combined of both pathways (*n* = 5). **(A,C)** The bars indicate the median. **(B,D–H)** Lines between dots indicate paired samples. Data are related to those of the control cultures without CAFs. Wilcoxon matched-pairs signed rank test was used to detect statistically significant differences between paired samples in the absence or presence of CAFs, and between cultures with isotype or vehicles and cultures with the different immunomodulatory factors neutralized, *P < 0.05, ***P < 0.001, ns, not significant.

To study how CAFs affect the proliferative response of T-cells, CFSE-labeled PBMCs from healthy donors were cultured in the presence or absence of irradiated patient-derived CAFs and stimulated or not with OKT3 for 5 days. The presence of CAFs significantly reduced the proliferation of CD4^+^ (*p* < 0.0001) and CD8^+^ (*p* < 0.0001) T-cells (Figure 2A). This effect was mediated in a dose-dependent manner (Supplementary Figure S2A). T-cell proliferation was not induced by CAFs alone (Figure 2A). To clarify whether the MHC mismatch between the PBMCs and CAFs is affecting the assay, a number of experiments were done with autologous PBMCs. The same effect was seen when PBMCs from patients were co-cultured with autologous CAFs derived from the same patients (Figure 2B).

To determine whether the observed immunosuppressive effects of CAFs were mediated in a contact dependent or independent fashion, CFSE-labeled PBMCs were cultured in direct contact with CAFs or separated by a transwell membrane. As shown in Figure 2C, no significant differences were found in the level of inhibition of CD4^+^ and CD8^+^ T-cells between the two conditions, indicating that the suppression is mainly mediated by soluble factors.

To examine whether the immunomodulatory properties of CAFs derived from PDAC differed from CAFs isolated from colloid carcinoma and adenosquamous carcinoma (Table 1), we co-cultured the different types of CAFs paired with the same PBMC donors. The CAFs derived from the other types of pancreatic cancer displayed a similar effect on T-cell proliferation and co-inhibitory markers expression as observed for the CAFs isolated from PDAC (Supplementary Figures S3A–E).

### COX-2 Is Involved in the Immune Regulatory Functions of Pancreatic CAFs

Next, we investigated whether the immune suppressive molecules PGE_2_, TGF-β, IDO, and PD-L1/L2, that previously have been described to be expressed by carcinoma fibroblasts (23–25), were involved in pancreatic CAF-mediated suppression. By inhibiting PGE_2_ production with the COX-2 inhibitor indomethacin, CD4^+^ and CD8^+^ T-cell proliferation was partially restored in all paired samples (*n* = 13) (Figure 2D). Blockade of the PD-1 pathway with anti-PD-L1 and PD-L2 antibodies significantly restored CD4^+^ T-cell proliferation and was restored in over half of the experiments for CD8^+^ T-cells (11 out of 17) (Figure 2E). We found no correlation between efficacy of PD-L1 and PD-L2 blockade and the expression levels of these proteins in CAFs (data not shown). By blocking TGF-β with neutralizing anti-TGF-β antibodies or IDO with 1-D/L-MT, T-cell proliferation was not significantly restored (Figures 2F,G). To examine if the combined blocking of the COX-2 and PD-1 axis would further abrogate the CAF-mediated suppression on T-cells, we performed a set of experiment using indomethacin, anti-PD-L1/L2 antibodies or both. Again, COX-2 inhibition increased the proliferation for CD4^+^ and CD8^+^ T-cells (median 2.7-fold and 1.8-fold, respectively), whereas blockade of PD-L1 and PD-L2 only marginally increased the proliferation (median 1.2-fold for CD4^+^ and 1.1-fold for CD8^+^) (Figure 2H). When combining inhibition of both the COX-2 and PD-1 axis, no increased effect on T-cell proliferation was observed (Figure 2H). Overall, since one of the main functions of the COX-2 inhibitor indomethacin is to block PGE_2_ synthesis, our data suggests that PGE_2_ was one important factor involved in the CAF-mediated anti-proliferative capacity on T-cells. PD-L1 and PD-L2 probably also play a role in suppressing T-cell proliferation in this setting, although this pattern was only significant for CD4^+^ T-cells.

### Pancreatic CAFs Upregulate the Expression of FOXP3 in Unstimulated CD4^+^ T-Cells

CAFs from other types of cancers have previously been shown to induce regulatory T-cells (Tregs) (11, 26). Our results also showed a significant increase in FOXP3-expressing cells among CD4^+^ T-cells when PBMCs were cultured in the presence of CAFs without further stimulation. This was observed in both direct co-cultures (median 2.3%) and indirect transwell cultures (median 1.8%) conditions compared to PBMCs cultured alone (median 1 and 0.7%, respectively) (Supplementary Figure S4A). CAFs also induced a significant increase in IL-10 levels in the supernatants, which was most pronounced when cell-cell contact was allowed (Supplementary Figure S4B). CAFs alone did not produce IL-10, suggesting that PBMCs were the source of IL-10, but it is also possible that IL-10 could be produced by CAFs after receiving signals derived from PBMCs (Supplementary Figure S4B). No significant correlation was observed between the proportion of FOXP3^+^ in CD4^+^ T-cells and the levels of IL-10 in the supernatant (data not shown).

### Pancreatic CAFs Upregulate the Expression of Immune Checkpoints on Proliferating T-Cells

**Figure 3:**
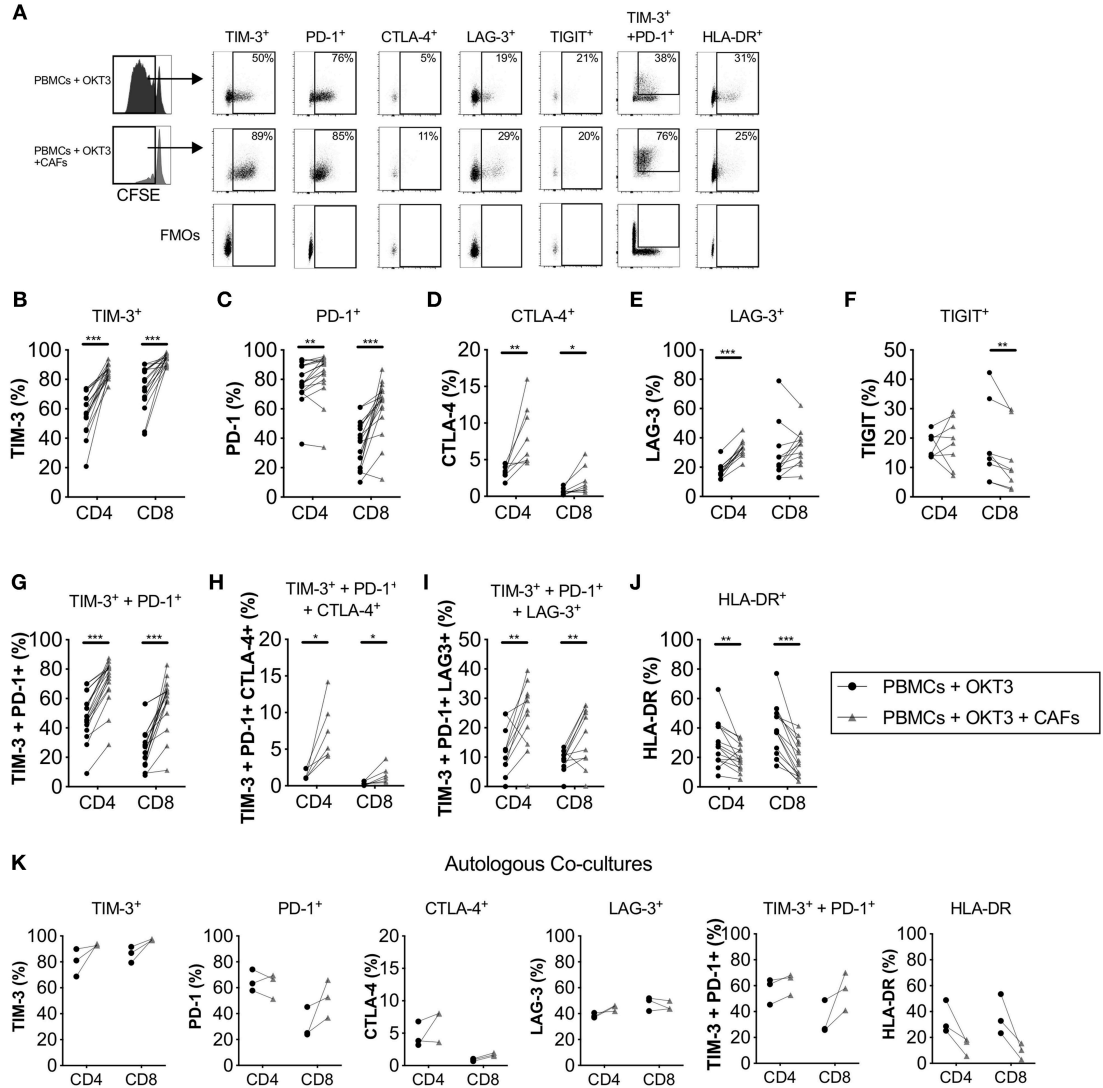
CAFs promote the expression of co-inhibitory markers on proliferating T-cells. CFSE-labeled PBMCs were co-cultured in the absence (•) or presence (▴) of CAFs and stimulated with OKT3 (25 ng/ml) for 5 days. **(A)** Representative flow cytometry dot plots on proliferating CD4^+^ T-cells showing the expression of different molecules after stimulation in the presence or absence of allogeneic CAFs. Expression of **(B)** TIM-3 (*n* = 18), **(C)** PD-1 (*n* = 18), **(D)** CTLA-4 (*n* = 8), **(E)** LAG-3 (*n* = 12), **(F)** TIGIT (*n* = 8), **(G)** co-expression of TIM-3 and PD-1 (*n* = 16), **(H)** co-expression of TIM-3, PD-1 and CTLA-4 (*n* = 6) and **(I)** co-expression of TIM-3, PD-1 and LAG-3 (*n* = 12), and **(J)** HLA-DR (*n* = 18) on proliferating CD4^+^ and CD8^+^ T-cells in the absence (•) or presence (▴) of allogeneic CAFs. **(K)** Co-inhibitory markers on patient-derived CD4^+^ and CD8^+^ T-cells in the absence or presence of autologous CAFs (*n* = 3). **(A–J)** The bars indicate the median. **(K)** Lines between dots indicate pared samples. Wilcoxon matched-pairs signed rank test was used to detect statistically significant differences, *P < 0.05, **P < 0.01, ***P < 0.001.

Since the tumor microenvironment promotes expression of co-inhibitory markers on T-cells, we next sought to examine if pancreatic CAFs are involved in altering the expression of these receptors. We investigated how CAFs affect the expression of five co-inhibitory receptors on activated proliferating T-cells. We found that the expression of TIM-3 (Figures 3A,B), PD-1 (Figures 3A,C) and CTLA-4 (Figures 3A,D) were higher in the presence of CAFs on both CD4^+^ and CD8^+^ T-cells in a dose-dependent manner (Supplementary Figures S2B–E) compared to the T-cells cultured in the absence of CAFs. The frequency of LAG-3 expression was increased in CD4^+^ T-cells (Figures 3A,E), while the levels of TIGIT expression was lower on CD8^+^ T-cells in the presence of CAFs (Figures 3A,F). The co-expression of TIM-3 and PD-1 (Figures 3A,G), TIM-3, PD-1 and CTLA-4 (Figure 3H) and TIM-3, PD-1, and LAG-3 (Figure 3I) were also higher in the presence of CAFs. Interestingly, the up-regulation of co-inhibitory markers did not coincide with a higher expression of the late activation marker HLA-DR, which was lower in the presence of CAFs on both CD4^+^ and CD8^+^ proliferating T-cells (Figures 3A,J) in a dose-dependent manner (Supplementary Figure S2E). The same tendency was observed when patient-derived PBMCs were co-cultured with autologous CAFs (Figure 3K).

The up-regulation of co-inhibitory markers on T-cells was at least partially mediated by soluble factors since no significant differences were found on the expression of TIM-3, LAG-3 and CTLA-4 on CD4^+^ or CD8^+^ T-cells in transwell cultures conditions compared to direct co-cultures (Supplementary Figures S5A,B,D–F). Additionally, no difference were observed for HLA-DR expression (Supplementary Figure S5G). Only PD-1 expression was lower on CD4^+^ and CD8^+^ T-cells in transwell cultures compared to co-cultures, although PD-1 expression was still higher compared to activated PBMC alone (Supplementary Figure S5C).

### Immunoregulatory Functions of CAFs and Pancreatic Tumor Cells in Combination

Next, we examined how CAFs and tumor cells in combination would affect T-cell proliferation and expression of co-inhibitory markers. Overall, as shown by the connecting lines, both CAFs and PANC-1 inhibited CD8^+^ T-cells proliferation (Supplementary Figure S6A) and upregulated the co-inhibitory markers PD-1, TIM-3 and LAG-3 compared to activated T-cells cultured alone (Supplementary Figures S6B–D). However, by using multiple comparison tests, there was a stronger inhibitory effect of CAFs on T-cells proliferation compared to PANC-1 cells (Supplementary Figure S6A). Moreover, the upregulation of the expression of the co-inhibitory markers TIM-3 and LAG-3 on proliferating T-cells was greater in the presence of CAFs compared to PANC-1 cells (Supplementary Figures S6C,D). Finally, the expression of HLA-DR was more inhibited in the presence of CAFs compared to PANC-1 (Supplementary Figure S6F).

When both CAFs and PANC-1 were added to the PBMC cultures, the immunomodulatory effects were enhanced, further inhibiting T-cell proliferation and upregulating PD-1 expression and the co-expression of PD-1 and TIM-3 on proliferating T-cells (Supplementary Figures S6A,B,D–F). This might suggest a possible crosstalk between CAFs and cancer cells that leads to an even stronger upregulation of inhibitory factors. However, further functional experiments are needed to confirm this.

### Localization of T-Cells and PD-1^+^ Cells in α-SMA^+^ Desmoplastic Tumor Stroma

**Figure 4:**
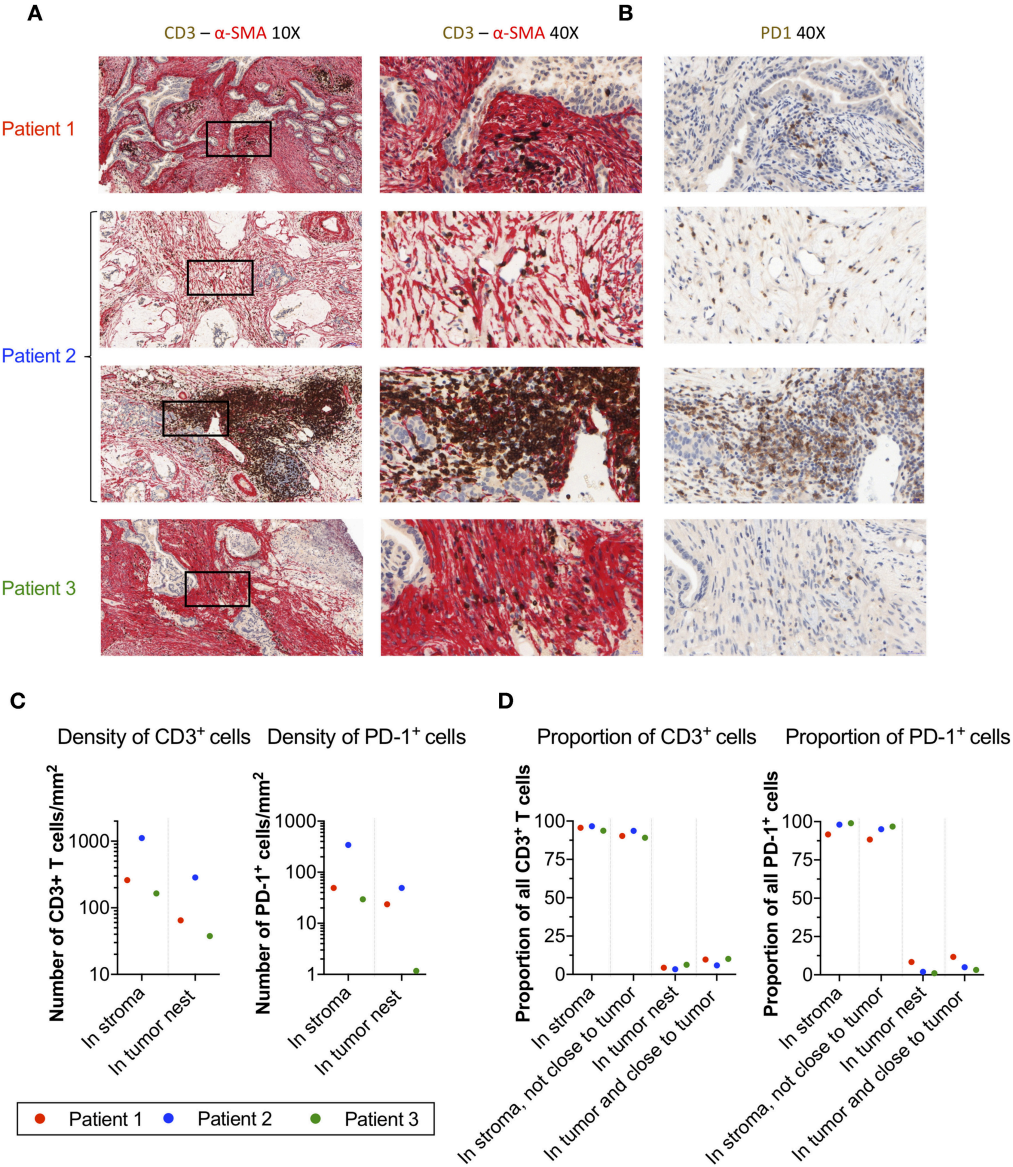
Localization of T-cells and PD-1^+^ cells in α-SMA^+^ desmoplastic tumor stroma. **(A)** Microphotographs of representative tumor areas from three different patients showing abundant desmoplastic stroma composed of α-SMA^+^ CAFs (red) with foci of CD3^+^ T-cells (brown). In these three patients, most of the T-cells were localized in the stroma with little direct cell-cell contact with adjacent adenocarcinoma cells. Second row of sample from patient 2 (a patient with colloid carcinoma of the pancreas) illustrates a peritumoral tertiary lymphoid structure. **(B)** Consecutive slides stained for PD-1 (brown) show abundant expression of PD-1 in T-cell rich areas within the desmoplastic stromal compartment. **(C)** Number of CD3^+^ T-cells and PD-1^+^ cells per mm^2^ in stroma and tumor nests in 4 annotations.**(D)** Percentage of CD3^+^ and PD-1^+^ cells detected in the stroma, in the stroma without close interaction with the tumor (20 μm), in the tumor nest, and in the tumor nest including positive cells in close proximity to the tumor (20 μm).

To corroborate the localization of PD-1^+^ T-cells in pancreatic tumors, we performed immunohistochemistry stainings of central tumor tissues from three donors, two with pancreatic ductal adenocarcinoma and one with colloid carcinoma of the pancreas. In all three cases, the tumor nests were surrounded by a dense α-SMA^+^ desmoplastic stroma (Figure 4A). T-cells were present in the tissues, but they were primarily found in the stroma with few T-cells infiltrating the tumor nests (Figure 4A). Stainings of consecutive slides further revealed that T-cells residing within the stroma expressed PD-1 (Figure 4B).

To quantify the number and location of the T-cells and PD-1^+^ cells within the tumor nests and stroma, we the used the QuPath software to analyze the immunohistochemistry stainings. A step-by-step description of the analysis is shown in Supplementary Figure S7. From each slide, we quantified the number of cells in four different areas. The data retrieved from the analysis is shown in Table 2. The proportion of CD3^+^ T-cells among total number of nucleated cells in these areas varied between 2.6 and 26%, and the proportion of PD-1^+^ cells between 0.5 and 2.1% (Table 2). When quantifying the number of T-cells and PD-1^+^ cells in tumor and stroma, we found that both T-cells and PD-1^+^ cells were more numerous per mm^2^ in the stroma compared to the tumor nests in all three patients (Figure 4C and Table 2). Consequently, a larger proportion of all CD3^+^ T-cells in the examined tissue was located to the stromal compartment, ranging between 94 and 96% of all detected T-cells (Figure 4D). Likewise, the percentage of all PD-1^+^ cells was also higher in the stroma compared to the tumor nests (Figure 4D). We next investigated if inclusion of T-cells in close proximity to the tumor cells (20 μm), which potentially could mediate anti-tumor effects, would change the delineation of the density T-cells in contact with tumor cells. As shown in Figure 4D, we found that including the cells in close proximity to tumor cells marginally changed the proportion of T-cells localized to the stroma. A very similar pattern was observed for PD-1 expressing cells (Figure 4D). These data suggest that the majority of T-cells in the pancreatic tumor microenvironment are trapped in the stromal compartment, with little interaction with tumor cells. Furthermore, PD-1 expressing cells were predominantly localized to the stroma, suggesting a role for CAFs in the upregulation of PD-1 also *in vivo*.

**Table 2:** Computational analysis of immunohistochemistry stainings.

	**Patient 1**	**Patient 2**	**Patient 3**
Number of annotations	4	4	4
Total number of nucleated cells^*^	22,753	15,537	20,447
	20,159	14,970	18,752
Total tumor area (mm^2^)^*^	0.62	0.48	0.90
	0.64	0.49	0.85
Total stroma area (mm^2^)^*^	3.4	3.5	3.1
	3.4	3.5	3.1
Number of CD3^+^ cells in total area	919	4,065	543
Number of PD-1^+^ cells in total area	180	1,226	94
Number of CD3^+^ cells per mm^2^ in tumor	65	285	38
Number of CD3^+^ cells per mm^2^ in stroma	260	1,115	164
Number of PD-1^+^ cells per mm^2^ in tumor	24	49	1
Number of PD-1^+^ cells per mm^2^ in stroma	49	342	29
Percentage CD3^+^ cells of all nucleated cells in total area (%)	4.0	26.2	2.7
Percentage PD-1^+^ cells of all nucleated cells in total area (%)	0.89	8.2	0.50
Percentage of CD3^+^ cells in tumor (%)	4.4	3.3	6.3
Percentage of CD3^+^ cells in and close to tumor (20 μm) (%)	9.7	5.9	10.1
Percentage of CD3^+^ cells in stroma (%)	95.6	96.7	93.7
Percentage of CD3^+^ cells in stroma, not close to tumor (20 μm) (%)	90.3	93.6	89.1
Percentage of PD-1^+^ cells in tumor (%)	8.3	2.0	1.1
Percentage of PD-1^+^ cells in and close to tumor (20 μm) (%)	11.7	5.0	3.2
Percentage of PD-1^+^ cells in stroma (%)	91.7	98.0	98.9
Percentage of PD-1^+^ cells in stroma, not close to tumor (20 μm) (%)	88.3	95.0	96.8

* *Indicates the number of cells or areas from the two consecutive slides (CD3 and PD-1, respectively)*.

### PGE_2_ Upregulates the Expression of Immune Checkpoints in Proliferating T-Cells

**Figure 5:**
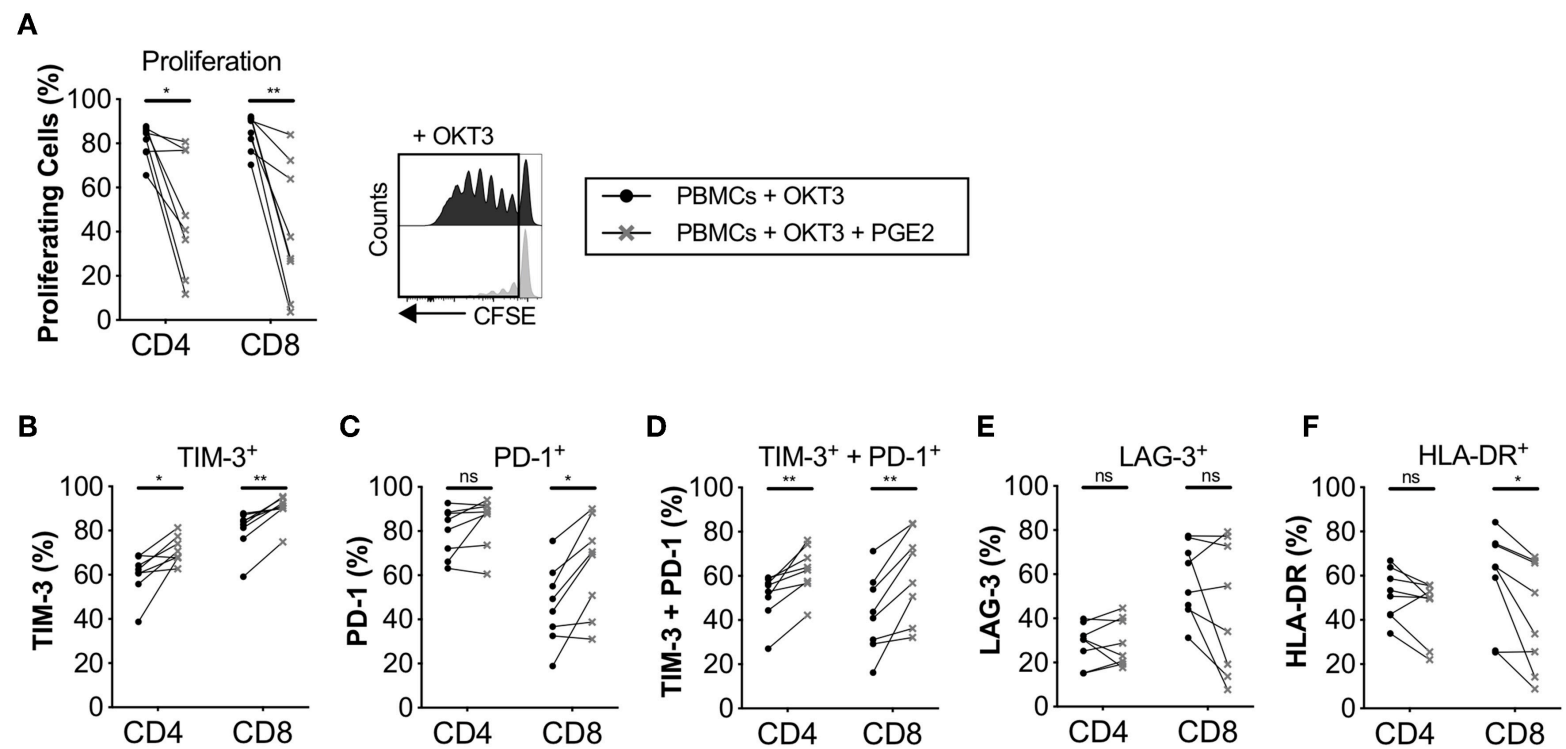
PGE2 inhibits T-cell proliferation and upregulate co-inhibitory markers on proliferating T-cells. CFSE-labeled PBMCs were cultured in the absence (•) or presence (**×**) of 0.1μg/ml PGE_2_ and stimulated with OKT3 (25 ng/ml) for 5 days (*n* = 8). **(A)** Frequency of proliferating CD4^+^ and CD8^+^ T-cells (left). Representative CFSE histograms on CD4^+^ T-cells (right). Expression of **(B)** TIM-3, (**C**) PD-1, **(D)** TIM-3 and PD-1 **(E)** LAG-3, and **(F)** HLA-DR on proliferating T-cells. Lines between dots indicate paired samples. Wilcoxon matched-pairs signed rank test was used to detect statistically significant differences *P < 0.05, **P < 0.01, ns (not significant).

Since we found that the immune modulatory activity of CAFs was at least partly mediated by COX-2 (Figure 2), we next examined the impact of PGE_2_ on T-cell proliferation and expression of co-inhibitory markers. The concentration of PGE_2_ used was similar as in previously reported studies (27). As previously shown by others (28), we found that PGE_2_ inhibits T-cell proliferation (Figure 5A), but we also found that it promoted the up-regulation of TIM-3 and PD-1 and consequently co-expression of TIM-3 and PD-1 on T-cells (Figures 5B–F). However, no effect on LAG-3 expression was observed (Figure 5E). PGE_2_ also decreased the expression of HLA-DR in CD8^+^ T-cells, but not significantly in CD4^+^ T-cells (Figure 5F). The median reduction in proliferation after addition of PGE_2_ was 1.7- and 2.7-fold for CD4^+^ and CD8^+^ T-cells, respectively, which can be put in comparison to CAF-mediated suppression of a median of 2.6-fold for CD4^+^ T-cells and 2-fold for CD8^+^ T-cells (Figure 2A).

### T-Cells Expressing Co-inhibitory Markers Are Less Functional in the Presence of CAFs

**Figure 6:**
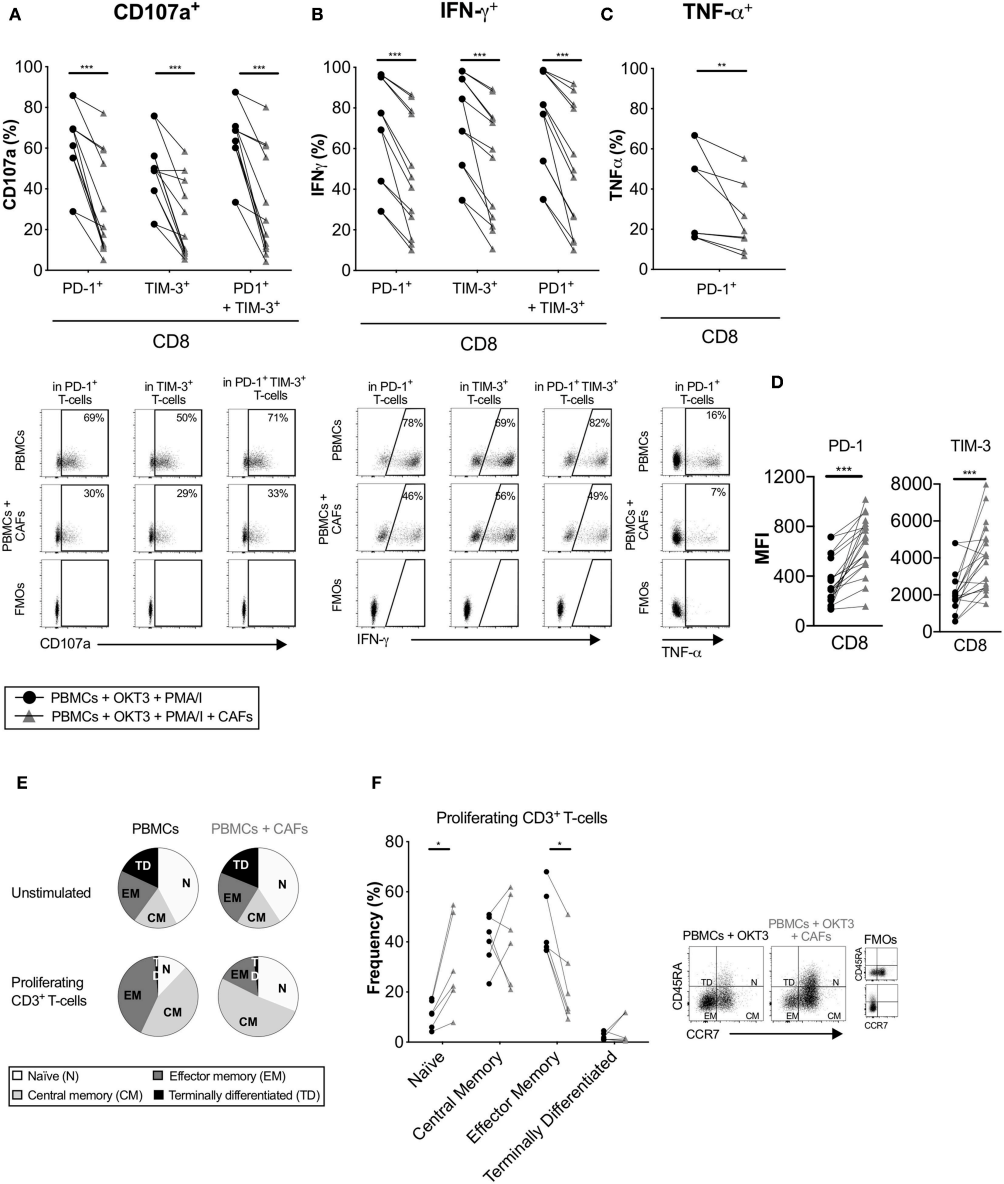
CAFs promote non-functional T-cells. CFSE-labeled PBMCs were co-cultured in the absence (•) or presence (▴) of CAFs and stimulated with OKT3 (25 ng/ml). On day 5 PBMCs were re-stimulated with PMA/Ionomycin (I) for 6 h. Expression of **(A)** CD107a and **(B)** IFN-γ in PD-1^+^, TIM-3^+^ and PD-1^+^TIM-3^+^ CD8^+^ T-cells (*n* = 12). **(C)** Expression of TNF-α in PD-1^+^ CD8^+^ T-cells (*n* = 8) (upper). Representative dot plots showing the gating strategy in **(A–C)** (bottom). **(D)** Median fluorescence intensity (MFI) of PD-1 and TIM-3 expression on CD8^+^ T-cells. **(E)** Pie charts with proportions of CD3^+^ T-cell memory subsets in unstimulated and proliferating T-cells after OKT3 stimulation. **(F)** Frequency of CD45RA^+^ CCR7^+^ (naïve), CD45RA^−^ CCR7^+^ (central memory), CD45RA^−^ CCR7^−^ (effector memory), CD45RA^+^ CDR7^−^ (terminally differentiated) expressed as percentage of proliferating CD3^+^ T-cells (*n* = 6) in the presence or absence of CAFs (left). Flow cytometry gating strategies for naïve (N), central memory (CM), effector memory (EM) and terminally differentiated (TD) on proliferating CD3^+^ T-cells (right). Lines between dots indicate paired samples. Wilcoxon matched-pairs signed rank test was used to detect statistically significant differences *P < 0.05, **P < 0.01, ***P < 0.001.

Co-inhibitory receptors are associated with T-cell exhaustion, but they can also indicate T-cell activation and differentiation. Having shown that CAFs upregulate the expression of co-inhibitory receptors on proliferating T-cells, we investigated whether CAFs alter the cytokine production of T-cells expressing PD-1 and/or TIM-3. After initial stimulation of T-cells in the presence or absence of CAFs for 5 days, T-cells were restimulated with PMA/Ionomycin for 6 h. Figure 6 shows that proliferating CD8^+^ T-cells expressing the co-inhibitory markers PD-1, TIM-3 or co-expressing both PD-1 and TIM-3 expressed less CD107a and IFN-γ in the presence of CAFs in all paired samples (Figures 6A,B). In addition, CD8^+^ T-cells expressing PD-1 contained lower levels of TNF-α (Figure 6C). The same pattern was also observed for CD4^+^ T-cells (data not show). Thus, this indicates that the CAPSC-mediated expression of co-inhibitory markers leads to loss of T-cell effector functions.

When we examined the intensity of expression of PD-1 and TIM-3 on proliferating T-cells, we found that CD8^+^ T-cells that had been cultured in the presence of CAFs had a higher median fluorescence intensity (MFI) expression of these markers (Figure 6D). Thus, this could suggest that the decreased functional effect observed above could be due to a higher intensity of expression of PD-1 and TIM-3.

We next examined the impact of CAFs on the memory phenotype of the proliferating CD3^+^ T-cells. After stimulation with OKT3 in the absence of CAFs, a large proportion of the T-cells differentiated into effector (CD45RA^−^CCR7^−^) or central (CD45RA^−^CCR7^+^) memory T-cells. In the presence of CAFs, a larger proportion of the T-cells retained their naïve phenotype (CD45RA^+^CCR7^+^) compared to T-cells cultured in the absence of CAFs, and a smaller proportion of the T-cells adopted an effector memory phenotype (Figures 6E,F). Similar results were found when using a transwell system (Supplementary Figure S5H). Together, these results suggest that CAF-derived soluble factors are able to suppress differentiation of effector memory T-cells, which may further explain the decreased functional T cell response.

### PD-1 Expression Is Negatively Correlated to the Proliferative Capacity of CD8^+^ T-Cells in the Presence of CAFs

**Figure 7:**
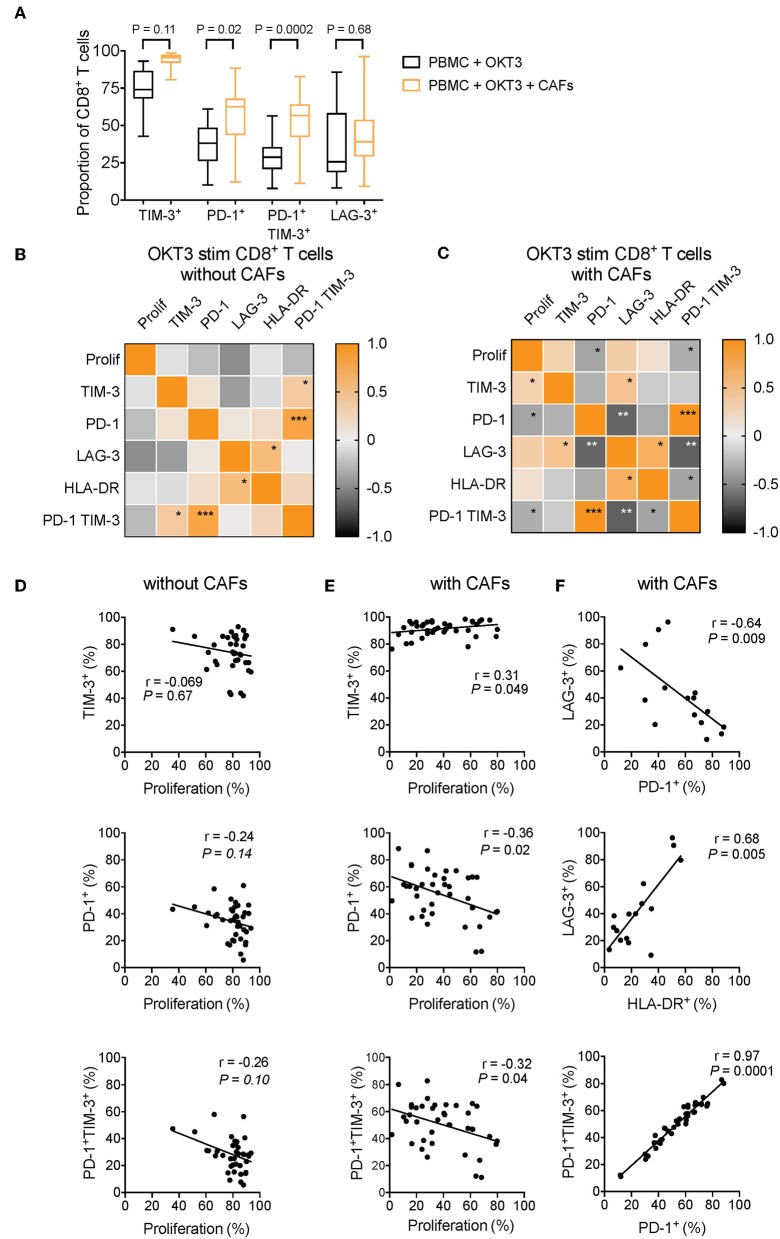
CAF-induced expression of PD-1 is negatively associated to CD8^+^ T-cell proliferation. **(A)** Multiple comparison test between expression of different co-inhibitory markers on CD8^+^ T-cells in the absence (black) or presence (orange) of CAFs after OKT3 stimulation (*n* = 40 for TIM-3 and PD-1, and *n* = 16 for LAG-3) in pooled experiments. **(B,C)** Heat map correlation matrix showing positive (orange) and negative (black) correlations between proliferation and co-inhibitory marker expression in OKT-3 stimulated CD8^+^ T-cells (*n* = 40 for proliferation, TIM-3 and PD-1, *n* = 16 for TIM-3, and *n* = 34 for HLA-DR) in the **(B)** absence or **(C)** presence of CAFs. **(D,E)** Correlations between proliferation and TIM-3, PD-1, and PD-1 and TIM-3 expression in the absence or presence of CAFs. **(F)** Correlation between expression of LAG-3 and expression of PD-1, and HLA-DR on T-cells cultured in the presence of CAFs. **(F)** Correlations between expression of PD-1 and co-expression of PD-1 and TIM-3 in CD8^+^ T-cells cultured in the presence of CAFs. Friedman's test followed by Dunn's test was used to evaluate significant difference between groups. Correlations were evaluated by using Spearman's correlation test. Spearmen *r* and *p-*values are presented.

To determine which co-inhibitory markers that was most strongly affected by CAFs, we performed a multiple comparison test on pooled data from different experiments. As shown in Figure 7A, co-expression of PD-1 and TIM-3 followed by expression of PD-1 were the markers that was most strongly increased after co-culture with CAFs. This suggests that among the markers we have studied here, PD-1 is the factor that is most affected by CAFs.

To investigate how the expression pattern of co-inhibitory markers affects proliferation of CD8^+^ T-cells, we used the pooled data from different experiments to generate a correlation matrix. As shown in the correlation heat maps depicted in Figures 7B–C, we found that the pattern looked different depending on whether CAFs had been added to the cultures or not. In the presence of CAFs, proliferation was negatively associated to the expression of PD-1 and to co-expression of PD-1 and TIM-3 (Figures 7C,E), but proliferation was not significantly correlated to these factors in the absence of CAFs (Figures 7B,D). In contrast, proliferative responses were positively correlated to TIM-3 in the presence of CAFs (Figures 7C,E), but not in CD8^+^ T-cells cultured without CAFs (Figures 7B,D). LAG-3 was not associated to proliferation, but there was a trend for a negative association between LAG-3 and proliferation in T-cells stimulated in the absence of CAFs (*r* = −0.47 and *P* = 0.07) (Figure 7B).

Furthermore, in CD8^+^ T-cells cultured in the presence of CAFs, we found that PD-1 expression and co-expression of PD-1 and TIM-3 was negatively correlated to LAG-3 expression (Figures 7C,F). The late activation marker HLA-DR was also negatively correlated to PD-1 and TIM-3 co-expression (*r* = −0.36, *p* = 0.035), and the same trend was seen for PD-1 expression (*r* = −0.33, *p* = 0.053) (Figure 7C). In line with this, LAG-3 expression was positively associated to HLA-DR expression (Figures 7C,F), which was evident also in the absence of CAFs (Figure 7B). This suggest that LAG-3 is associated with activation, rather than co-inhibition, under these experimental settings. Finally, the expression of PD-1 was correlated to PD-1^+^TIM-3^+^ T-cells, both in the presence and absence of CAFs (Figures 7B,C,F), indicating that the majority of PD-1^+^ T-cells also express TIM-3. Together, this points to a complexity in the function of co-inhibitory marker expression and T-cell proliferation, but also suggests that the CAF-mediated upregulation of PD-1 play a key role in suppressing CD8^+^ T cell proliferation. However, further functional assays are needed to confirm this.

## Discussion

Tumors have developed several strategies to escape anti-tumor immunity, including physical barriers and recruitment of immunosuppressive cells. In pancreatic cancer, the tumor microenvironment is dominated by a dense stroma created by activated CAFs, which mediate tumor growth and progression by producing extracellular matrix proteins, growth factors, chemokines, and cytokines. Numerous studies have explored the immunosuppressive properties of CAFs and their role in supporting tumor cell growth by favoring the presence of tumor promoting immune cells [reviewed in Ziani et al. (29)]. However, most studies have focused on the effect of CAFs on the innate immune cells, rather than on the adaptive immune response. Since T-cells are the dominant immune cell subset with a potential capacity to eradicate tumor cells, we have performed an in-depth examination of how CAFs affect the phenotype and activation status of T-cells. One of the most interesting findings was that CAFs induce the expression of the co-inhibitory markers TIM-3, PD-1, CTLA-4, and LAG-3 on activated T-cells upon stimulation. We further identified PGE_2_ as an inducer of co-inhibitory marker expression. In addition, T-cells expressing immune checkpoints produce less IFN-γ, TNF-α, and CD107a after restimulation when CAFs had been present, suggesting that CAPCSs promote T-cell exhaustion that leads to functional incapacity. We used OKT3 as a stimulation since it is commonly used to study T cell activation and due to that it is difficult to find a more physiological stimuli that universally activates T-cells from different donors.

The phenotype and function of CAFs can be altered during *in vitro* cultivation and expansion. However, fibroblasts from different anatomical sites of the body have been shown to have distinct transcriptional patterns even after *in vitro* expansion, indicating a positional memory of stromal cells (30). We compared the phenotype of CAFs throughout passages 1 and up to 3, but no apparent differences were observed. To limit the potential variation by the phenotype throughout the experiments, all the fibroblasts used in the study were in passage 3. The high expression of the cancer-associated markers FAP and podoplanin in normal skin fibroblasts, could suggest an upregulation of some markers through serial passaging. However, the activation marker α-SMA was only expressed in CAFs.

Pancreatic CAFs display heterogeneity and several subtypes of CAFs have been identified in pancreatic cancer. Öhlund et al. have described two dynamic subsets of pancreatic CAFs based on the expression of α-SMA (6). A subpopulation of α-SMA^high^ myofibroblasts were predominantly localized in close proximity with tumor cells, while α-SMA^low^ cells represented inflammatory CAFs associated with elevated expression of tumor promoting cytokines, including IL-6, and were located more distantly from the tumor cells. Biffi et al. further showed that tumor-derived TGF-β is involved in driving differentiation into myofibroblasts, whereas IL-1 signaling via JAK/STAT activation promoted inflammatory CAFs (7). TGF-β also counteracted the differentiation of inflammatory CAFs. Furthermore, Neuzillet recently demonstrated the presence of at least four different subpopulations of pancreatic CAFs, which can be distinguished based on the expression of periostin, podoplanin, and myosin-11, and were also associated to prognostic impact (8). Thus, it can be speculated that both pro-tumorigenic and anti-tumorigenic subtypes of CAFs are present in pancreatic cancer. The CAFs studied here are likely a combination of different CAF subpopulations, and there was also an inter-individual variation in the expression of the majority of the markers investigated, including α-SMA and podoplanin. Fur future studies, it would be interesting to examine how different subpopulations of CAFs affect T-cell responses.

The binding interaction between PD-1, expressed on activated T-cells, and its co-regulatory ligands PD-L1 and PD-L2 has previously been suggested as a mechanism underlying the suppressive effects of fibroblasts (11, 25, 31, 32). Moreover, Shibuya et al. reported that the majority of TILs in pancreatic cancer express PD-1 (33), suggesting that PD-L1/PD-1 interactions can modulate T-cell cytotoxic functions in this malignancy (34). PD-L1 overexpression has been reported in PDAC and it has also been correlated with poor overall survival outcome (34–36). However, few studies have explored the expression PD-L1 and PD-L2 in human carcinoma fibroblasts. In our study, we show by flow cytometry that CAFs constitutively express PD-L1 and PD-L2 in a higher frequency than normal skin fibroblasts and that the expression varies greatly between patients. We further noted that PD-L2, rather than PD-L1, was the dominant PD-1 ligand. Nazareth et al. also showed abundant levels of PD-L1 and PD-L2 in a subset of fibroblasts obtained from non-small cell lung carcinoma (32), and another study by Takahashi et al. showed a modest, but constitutive, expression of these ligands in fibroblasts from head and neck squamous cell carcinoma (11). We found that blockade of these ligands partially restored the proliferative ability of CD4^+^ T-cells, which was also observed for CD8^+^ T-cells in 65% of the experiments. Studies by Nazareth et al. and Takahashi et al. could also restore the T-cell suppression in some samples. The difference in the expression and function of PD-L1 and PD-L2 within a tumor and between tumors further strengthen the general perception that fibroblasts are a diverse group of cells and its functional activity might vary depending on the tumor microenvironment and the cytokine milieu.

Even though the CAFs secretome is still not completely characterized, there is evidence that the wide variety of growth factors and cytokines released by CAFs play a major role in orchestrating tumor progression and immune evasion (4). Since our transwell assays indicated that the immunosuppressive effects of CAFs are mediated by soluble factors, we attempted to identify factors involved in T-cell suppression by blocking the activity of the known soluble immunosuppressive factors PGE_2_, TGF-β, and IDO. PGE_2_, the major metabolite of the COX-2 enzyme, is known for its role to promote tumor growth, survival, invasion, angiogenesis, and suppression of anti-tumor immunity (37, 38). However, little is still known about the importance of PGE_2_ in the context of CAF–mediated immunosuppression. The expression of COX-2 in fibroblasts from pancreatic cancer (23, 39) and other tumor types (40, 41) has been previously reported. Here, we show that by blocking PGE_2_ production with indomethacin, T-cell proliferation was partially restored and the positive effect was consistent in all paired samples. No increased effect in proliferation was observed when both the COX-2 and the PD-1 axis was blocked, suggesting that the COX-2 inhibition is the most important pathway. However, there is also a possibility that the CAFs upregulate even higher levels of the PD-1 ligands during the *in vitro* culture, which could reduce the efficacy of PD-1 axis blockade using neutralizing antibodies. PD-L1/PD-L2 knock-out experiments could be a better approach to fully explore the importance of the PD-1 axis in CAF-mediated T-cell suppression.

IDO and TGF-β are also expressed by CAFs (4, 24) and they can contribute to immune evasion by catabolizing tryptophan into a number of downstream immunosuppressive metabolites (42) and by targeting cytotoxic T-cell functions (43), respectively. However, T-cell proliferation was not affected by blocking these molecules in our system. This does not exclude the possibility that these molecules exert a negative regulatory activity *in vivo*. Fibroblast-derived soluble factors can modulate the tumor microenvironment by recruiting other immune cells, which in turn secrete more immunosuppressive cytokines, creating autocrine and paracrine loops which can interfere with T-cell activity (29, 44). Here, in line with previous studies we found that the proportion of CD4^+^FOXP3^+^ putative Tregs was significantly increased when CAFs were added to unstimulated PBMCs. This was also accompanied by increased levels of the immunosuppressive cytokine IL-10 in the supernatants, but the source of IL-10 remains to be determined. An association between tumor infiltrating FOXP3^+^ Tregs and a dismal prognosis has been shown in several cancers. Kinoshita et al. demonstrated that Tregs were primarily localized to the stroma in lung adenocarcinoma, and less to the tumor nest, and that CAFs isolated from tissues with high Treg numbers had a better FOXP3-inducing effect compared to CAFs from low Treg adenocarcinomas (26).

There is evidence that CAFs interact with T-cells in the tumor stroma by skewing the immune response toward Th2 (45), and cross-present antigens which leads to T-cell apoptosis and dysfunction (31). Studies with murine models have also reported that CAFs limit the access of CD8^+^ T-cells to the tumor (19, 46). However, this perception has been challenged by studies of stained human tissues samples which show a great variability of TILs and that the stroma microenvironment does not affect cytotoxic T-cell infiltration (15, 33). Stromnes et al. recently showed that the majority of T-cells localize to tertiary lymphoid structures rather than the tumor nest in many patients with high T-cell infiltration (47), which is in line with our findings that the majority of the T-cells reside in the desmoplastic stroma and that few T-cells are in immediate contact with cancer cells. Since a recent paper suggested that the desmoplastic stroma does not prevent T-cells from entering the tumor nests (15), we analyzed the distribution of T-cells and PD-1 expressing cells and also included cells that was in close proximity to the tumor cells. Although the number of patients analyzed are low, the data consistently shows that over 90% of all T-cells in the tumor microenvironment are localized to the stroma, and that inclusion of T-cells that are close enough to potentially interact with tumor cells only marginally increase the estimated T-cell density in tumors.

Regardless of the existence of TILs in pancreatic cancer and the fact that high number of TILs correlates with an increased overall survival (15, 16, 19), the prognosis is still dismal (1). This can partly be explained by that CAFs, together with tumor-infiltrating Tregs, M2 macrophages, and myeloid derived suppressor cells, create an immunosuppressive tumor milieu that inhibits the activation and function of effector T-cells (48, 49). Here, we have further explored the interactions between T-cells and CAFs and show, for the first time to our knowledge, that CAFs promote the expression of the co-inhibitory markers TIM-3, PD-1, CTLA-4 and LAG-3 on activated T-cells. Since CAFs markedly suppressed the proliferation of T-cells, we reasoned that the most fair way to compare the T-cell phenotypes would be to only study T-cells that respond to the stimulation, e g proliferating cells. In a more physiological setting, this would only apply to T-cells that have gone through clonal expansion after encountering their antigen. Our findings from the immunohistochemistry stainings of tumor tissue also suggest that PD-1^+^ T-cells can be found in close proximity to tumor cells but also to α-SMA^+^ CAFs. Thus, considering the high levels of PD-L1/L2 on CAFs, one could speculate that CAFs effect T-cell functionality *in vivo* upon PD-L1/L2 binding.

It has previously been proposed that the co-expression of inhibitory receptors rather than individual expression is indicative of T-cell exhaustion (50). We found a 5.9-fold and a 2.7-fold increase in the frequency of CD4^+^ and CD8^+^ T-cells, respectively, co-expressing TIM-3, PD-1 and CTLA-4 and a 2.3-fold and a 1.7-fold increase in the frequency of CD4^+^ and CD8^+^ T-cells, respectively, co-expressing TIM-3, PD-1 and LAG-3. We also observed that the proliferating T-cells positive for PD-1, TIM-3, or double positive for PD-1 and TIM-3 expressed lower levels of the degranulation marker CD107a and reduced levels of the inflammatory cytokines IFN-γ and TNF-α when CAFs had been present. The low cytokine production could also be explained by the fact that most of the proliferating T-cells retain their naïve phenotype in the presence of CAFs. Thus, although some T-cells differentiate into a central memory phenotype, they fail to fully differentiate into cytotoxic effector memory T-cells. We used the expression of CD107a as a marker for T-cell degranulation, which previously have been shown to be a good surrogate marker for cytolytic activity (51). However, it still needs to be determined if CAFs diminishes the capacity to kill tumor cells in an antigen-specific manner. Considerable research has been devoted to investigate how tumor cells achieve immune evasion by interacting with T-cell co-inhibitory markers (52). However, less attention has been paid on the impact of the tumor stroma fibroblasts on T-cell functions. It remains to be determined if our findings that CAFs are capable to disarm T-cell effector functions and promote upregulation of co-inhibitory markers are also relevant *in vivo*.

Among the co-inhibitory markers that we have studied here, PD-1 and co-expression of PD-1 and TIM-3 were most strongly affected by pancreatic CAFs. Correlation analyses further indicated that PD-1 expression and co-expression of PD-1 and TIM-3 were negatively associated to proliferative capacity of CD8^+^ T-cells that had been cultured with CAFs. On the other hand, TIM-3 and LAG-3 expression was not associated to less proliferation, but rather to increased activation as determined both by HLA-DR expression and proliferation. Thus, the function of co-inhibitory markers is complex in activated T-cells, but it highlights an important role for PD-1 expression in the CAF-mediated suppression of T-cells.

We used allogeneic PBMC in the majority of the experiments, and one could argue that the MHC-mismatch between the PBMCs and CAFs could affect the response of the assay. However, very similar results were observed in transwell settings, suggesting that response to alloantigens are not affecting the assay. This also was confirmed in a number of experiments using autologous patient-derived PBMCs.

It has been reported that the expression of PD-1 on TILs is correlated with increased PGE_2_ levels in lung cancer (27). Since we showed that COX-2 inhibitors partially restored T-cell proliferation, we investigated whether adding PGE_2_ to PBMCs cultures induced co-inhibitory molecules on proliferating T-cells. Indeed, we observed that PGE_2_ inhibits T-cell proliferation and upregulates the expression of both PD-1 and TIM-3 on proliferating T-cells. These results are in line with previous studies showing that exhausted cytotoxic T-lymphocytes upregulate the expression of PGE_2_ receptors EP2 and EP4 (53) and that the combined inhibition of PD-1 signaling pathway and PGE_2_ increased the number and the cytotoxic effects of CD8^+^ T-cells (53). Interestingly in this context, COX-2 inhibitors have been shown to inhibit pancreatic stellate cell proliferation and activation (54). However, treatment with the COX-2 inhibitor celecoxib in combination with standard chemotherapy for pancreatic cancer did not show any clinical benefits (55). On the other hand, others have shown that inhibition of COX2 potentiates the effect of anti-PD-1 blockade in experimental models of melanoma, indicating that COX inhibitors could function as an adjuvant for immune checkpoint inhibition (38). Since it has been documented that T-cells tend to upregulate other co-inhibitory markers after PD-1 blockade, notably TIM-3 (56), our findings that PGE_2_ upregulate TIM-3 further strengthen the clinical relevance of combining COX inhibitors with anti-PD-1 treatment.

In order to find a successful cure for PDAC, it will be important to delineate the mechanisms behind the functional defects of TILs. It will likely involve several different treatment approaches, and our findings suggest that it is important to investigate the complex interactions between the CAFs and T-cells. An increased understanding of how fibroblasts from tumor tissues affect T-cell phenotype and functions could lead to the development of improved combined immunotherapy strategies.

## Ethics Statement

All subjects gave written informed consent in accordance with the Declaration of Helsinki. The protocol was approved by the board of ethics in research of Karolinska Institutet, entry nos. 2009/418-31/4, 2013/977-31.3, and 2017/722-32.

## Author Contributions

LG designed, performed, and analyzed the experiments, interpreted the results, and wrote the manuscript. CF analyzed the immunohistochemistry stainings and interpreted the results. PB performed digital analyses of the immunohistochemistry stainings and interpreted the results. KK and IS performed and analyzed the experiments. QM, and ER interpreted the results. HK conceived, designed, and analyzed the experiments, interpreted the results and wrote the manuscript. All authors participated in the final approval of the manuscript.

### Conflict of Interest Statement

The authors declare that the research was conducted in the absence of any commercial or financial relationships that could be construed as a potential conflict of interest.
