# Supplementary material for: Human Pancreatic Carcinoma-Associated Fibroblasts Promote Expression of Co-inhibitory Markers on CD4+ and CD8+ T-Cells
Source: Front Immunol. 2019 Apr 24;10:847. doi: 10.3389/fimmu.2019.00847 (PMC6491453; doi:10.3389/fimmu.2019.00847)
Supplement: Supplementary file 1 [file Data_Sheet_1.pdf]

Supplementary Table S1

Markers	Fluorochrome	Clone	Company	Catalogue number	Staining
**CAPSCs markers**					
CD29	PE	MAR4	BD	561795	EC
CD31	FITC	WM59	BD	555445	EC
CD44	FITC	G44-26	BD	560977	EC
CD49d	BV421	9F10	BD	565277	EC
CD73	PE	AD2	BD	550257	EC
CD105	PE	266	BD	560839	EC
CD106 (VCAM-1)	FITC	51-10C9	BD	551146	EC
CD54 (ICAM-1)	PE	HA-58	BD	555511	EC
HLA-I	FITC	G46-2.6	BD	560965	EC
HLA-DR	V500	G46-6	BD	561223	EC
PD-L2	APC	MIH18	BD	557926	EC
CD86	FITC	FUN-1	BD	555657	EC
CD90	PE	5E10	BD	561970	EC
EPCAM	FITC	9C4	BioLegend	324203	EC
PD-L1	PE	29E.2A3	BioLegend	329706	EC
Podoplanin	A647	NC-08	BioLegend	337007	EC
FAP	APC	427819	Bio-techne		EC
αSMA	A488	1A4	ABCAM	Ab184675	IC
-	7AAD	-	BD		
**Lymphocytes markers**					
CD3	PE-Cy-7	UCHT1	BD	563423	EC
CD3	V450	UCHT1	BD	560365	EC
CD4	A700	RPA-T4	BD	557922	EC
CD4	V500	RPA-T4	BD	560768	EC
CD8	APC-Cy7	SK1	BD	557834	EC
CD25	BV421	MA-251	BD	562442	EC
PD-1	BV421	EH12.1	BD	562516	EC
CD45RA	PE-Cy7	HI100	BD	560675	EC
CCR7	PECF594	150503	BD	562381	EC
CD152 (CTLA-4)	PE	BNI3	BD	555853	EC
TIGIT	A700	741182	BD	FAB7898N	EC
HLA-DR	V500	G46-6	BD	561223	EC
CD107a	PE	H4A3	BD	555801	EC
TIM-3	APC	F38-2E2	Miltenyi	130098936	EC
CD223 (LAG-3)	PE	REA351	Miltenyi	130105452	EC
FOXP-3	PE	235A/E7	eBioscience	12477742	IC
TNF-α	APC	MAb11	BD	554514	IC
IFN-γ	PE-Cy7	4S.B3	BD	557844	IC
-	7AAD	-	BD	559928	

Abbreviations; Markers; **CD**, Cluster of differentiation, **VCAM-1**, Vascular cell adhesion molecule-1, **ICAM-1**, Intracellular adhesion molecule, **HLA-I**, Human leukocyte antigen-I, **HLA-DR**, Human leukocyte antigen-antigen D Related, **EPCAM**, Epithelial cell adhesion, **PD-L** Programmed death-ligand, **FAP**, Fibroblast activation protein, **αSMA**, Alpha smooth muscle actin, **CCR7**, Chemokine receptor type 7, **CTLA-4**, Cytotoxic T lymphocyte-associated antigen 4, **TIGIT**, T cell immunoreceptor with Ig, **TIM-3**, T-cell immunoglobulin and mucin-domain containing-3, **LAG-3**, Lymphocyte activation gene, **FOXP-3**, Forkhead box-3, **TNF-α** Tumor necrosis factor alpha, **IFN-γ**, Interferon

gamma. Fluorochromes; **PE**, Phycoerythrin, **FITC**, Fluoresceinisothiocyanate, **BV421**, Brilliant violet 421, **V500**, Violet 500, APC, Allophycocyanine, **A647**, Alexa Fluor 647, **A488**, Alexa Fluor 488, **7-AAD**, 7-Aminoactinomycin D, **Pe-Cy7**, Phycoerythrin-cyanine 7, **V450**, Violet 450, **A700**, Alexa Fluor 700, **APC-Cy7**, Allophycocyanine-indo tricarbocyanine, **PE-CF594**, Phycoerythrin-CF 594. Companies; **BD**, BD Biosciences (Franklin Lakes, NJ, USA), **BioLegend**, Biolegend (San Diego, CA, USA), **Bio-techne**, (Mineapolis, MN, USA), **Abcam,** Abcam (Cambridge,UK), **Miltenyi**, Miltenyi Biotec (Bergisch Gladbach, Germany), **eBioscience**, eBioscience, (San Diego, CA, USA). Stainings; **EC**, Extracellular, **IC**, Intracellular.

Supplementary Figure S1

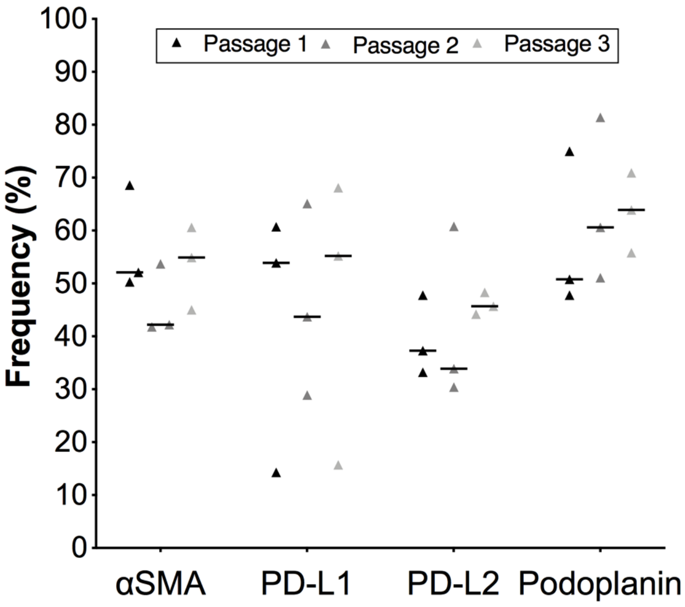

Supplementary Figure S1. Phenotypic analysis of carcinoma associated pancreatic fibroblasts throughout different passages. Comparison of α–SMA, PD-L1, PD-L2, and podoplanin expression between passage 1 (▲) (*n* = 3), 2 (▲) and 3 (▲) (*n* = 6). Bars show median. Wilcoxon matched-pairs signed rank test was used to detect statistically significant between passage 2 and 3.

Supplementary Figure S2

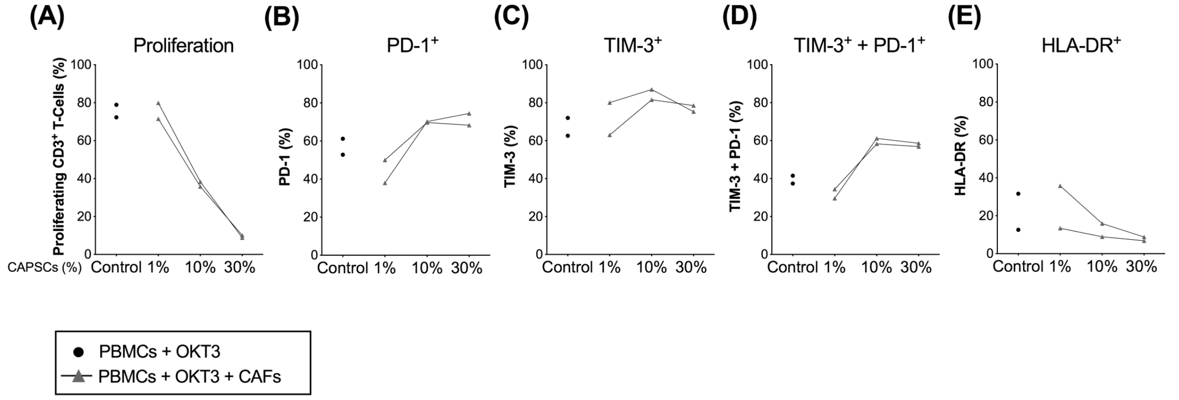

Supplementary Figure S2. The immunoregulatory effects of CAFs on T-cells are dose-dependent. CFSE-labeled PBMCs were co-cultured with indicated proportions of CAFs (▲) for 5 days or without CAFs (●) and stimulated with OKT3 (25ng/ml). On day 5, PBMCs were harvested and levels of **(A)** proliferating $CD3^+$ T-cells and the expression of **(B)** PD-1, **(C)** TIM-3, **(D)** TIM-3 and PD-1 and **(E)** HLA-DR on proliferating $CD3^+$ T-cells were measured by flow cytometry (n = 2).

Supplementary Figure S3

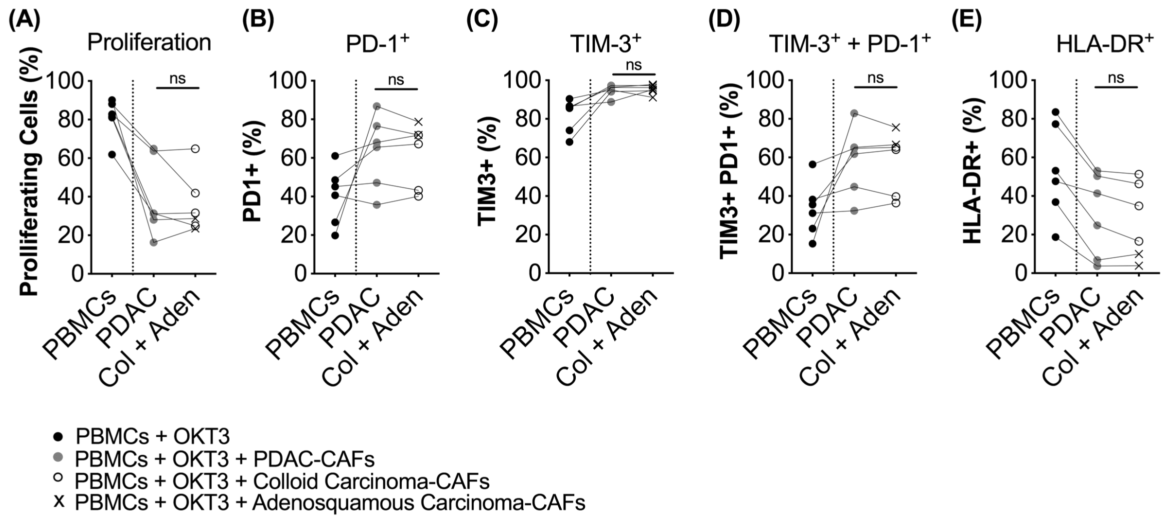

Supplementary Figure S3. CAFs derived from other types of pancreatic cancer display similar effects on T cells as PDAC-derived CAFs. CFSE-labeled PBMCs were co-cultured with CAFs derived from different types of pancreatic cancer; PDAC (●), colloid carcinoma (○) or adenosquamous carcinoma (×). **(A)** Frequency of proliferating CD8^{+} T cells. Expression of **(B)** PD-1 **(C)** TIM-3 **(D)** co-expression of PD-1, TIM-3 and **(E)** HLA-DR. Wilcoxon matched-pairs signed rank test was used to detect statistically significant difference; ns (not significant).

Supplementary Figure S4

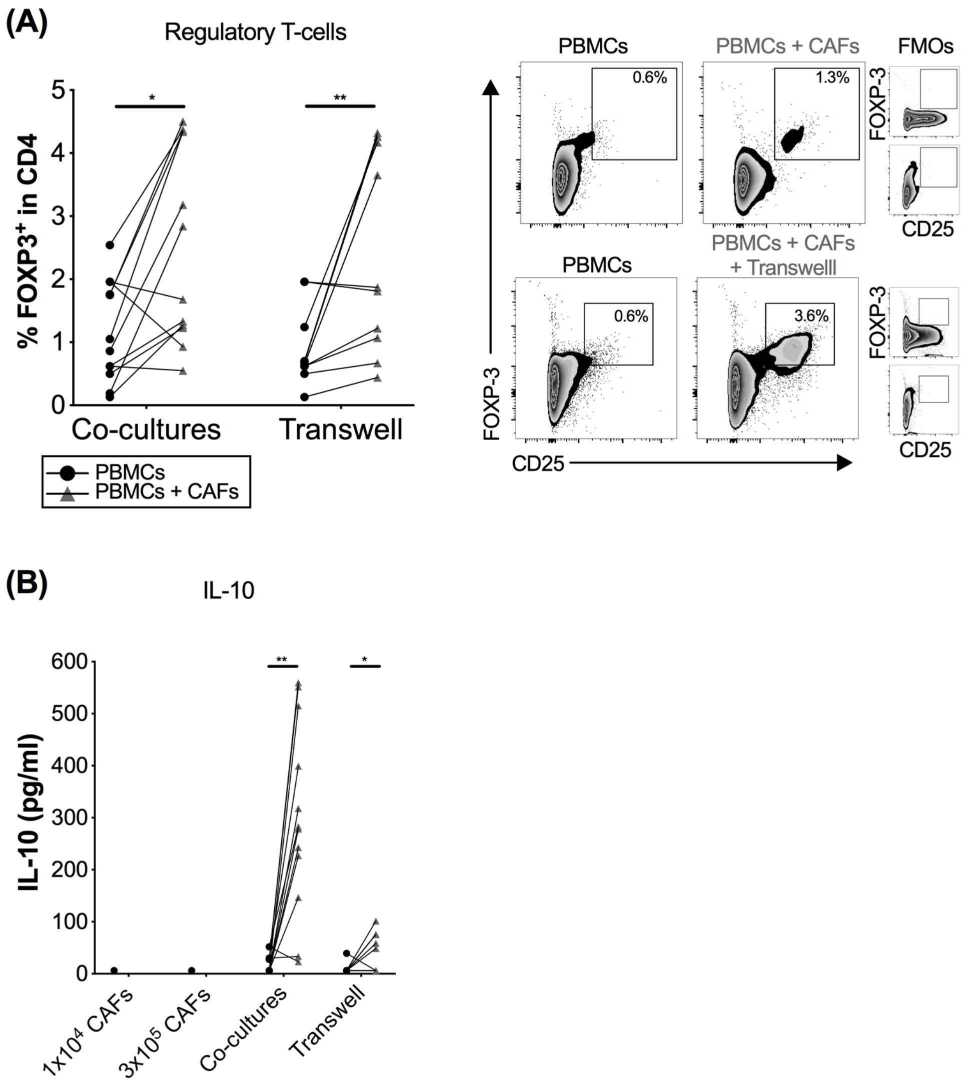

Supplementary Figure S4. CAFs promote regulatory T-cells both in direct co-cultures and transwell cultures in unstimulated conditions. PBMCs were co-cultured in the absence or presence of CAFs in direct co-cultures or in indirect transwell cultures and left unstimulated for 5 days. **(A)** (right) Proportion of FOXP3$^+$CD25$^+$ in CD4$^+$ T-cells in the absence (●) or presence of CAFs (▲) in co-cultures (n = 12) and transwell conditions (n = 10). (left) Representative dot plots showing the gating strategy. **(B)** Levels of IL-10 measured with ELISA in co-cultures (n = 12) and transwell supernatants (n = 10) after 5 days. Dots and lines show paired samples. Wilcoxon matched-pairs signed rank test was used to detect statistically significant difference $*P < 0.05$ $**P < 0.01$.

Supplementary Figure S5

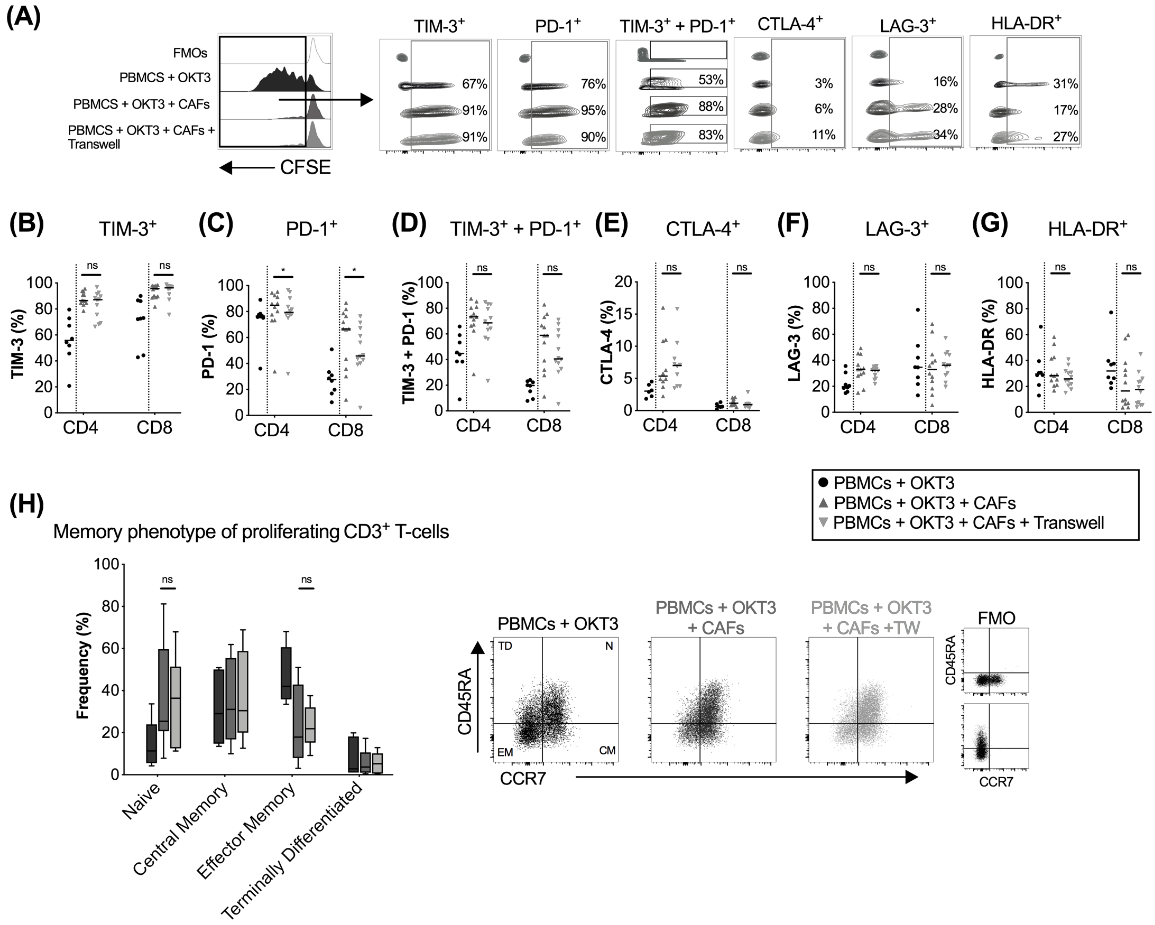

Supplementary Figure S5. The immunoregulatory effects of CAFs are cell contact independent. **(A)** Representative flow cytometry dot plots on proliferating CD4$^+$ T-cells showing the expression of co-inhibitory markers after stimulation in the presence or absence of CAFs in direct or transwell cultures. **(B-G)** Expression of **(B)** TIM-3 (n = 12), **(C)** PD-1 (n = 20), **(D)** co-expression of TIM-3 and PD-1 (n = 12), **(E)** CTLA-4 (n =10), **(F)** LAG-3 (n = 12), and **(G)** HLA-DR (n = 12) on proliferating CD4$^+$ and CD8$^+$ T-cells in the absence of CAFs (●) or presence of CAFs in direct (▲) or transwell (▼) cultures conditions. **(H)** (left) Frequency of CD45RA$^+$ CCR7$^+$ (naïve), CD45RA$^-$ CCR7$^+$ (central memory), CD45RA$^-$ CCR7$^-$ (effector memory), CD45RA$^+$ CDR7$^-$ (terminally differentiated) expressed as percentage of proliferating CD3$^+$ T-cells (n = 8) in the absence of CAFs (■), in direct (■) or in transwell (■) cultures conditions (n = 8). (right) Flow cytometry gating strategies for naïve (N), central memory (CM), effector memory (EM) and terminally differentiated (TD) proliferating CD3$^+$ T-cells. **(B-G)** Bars and **(H)** box and whiskers show the medians. Wilcoxon matched-pairs signed rank test was used to detect statistically significant difference *P < 0.05, ns (not significant).

Supplementary Figure S6

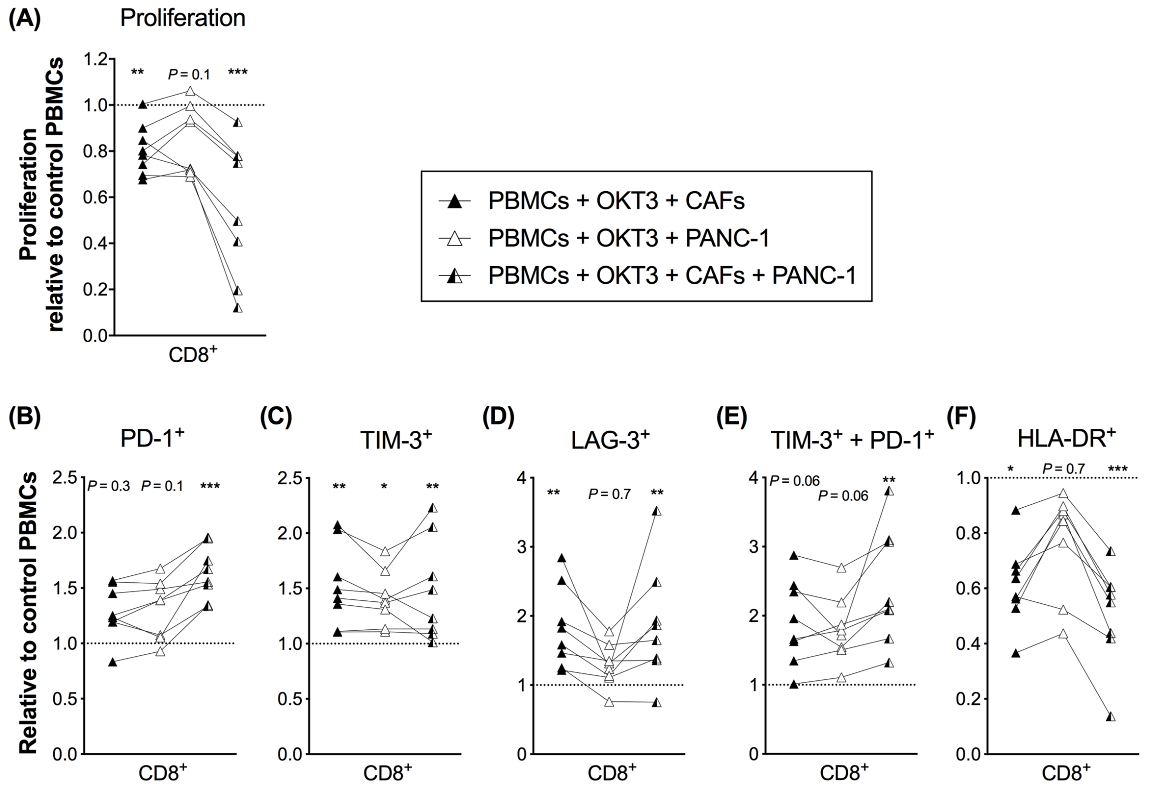

Supplementary Figure S6. Immunoregulatory functions of CAFs and pancreatic tumor cells in combination. CFSE-labeled PBMCs were co-cultured in the absence or presence of (▲) CAFs, (△) PANC-1 or (△) both CAFs and PANC-1 and stimulated with OKT3 (25ng/ml) (*n* = 8). **(A)** Frequency of proliferating T-cells. Expression of **(B)** PD-1, **(C)** TIM-3, **(D)** LAG-3, **(E)** co-expression of PD-1 and TIM-3, and **(F)** HLA-DR. Friedman's test with a Dunn's post test was used to detect statistically significant difference between the three different co-cultures and PBMCs cultured alone *$P < 0.05$, **$P < 0.01$, ***$P < 0.001$.

Supplementary Figure S7

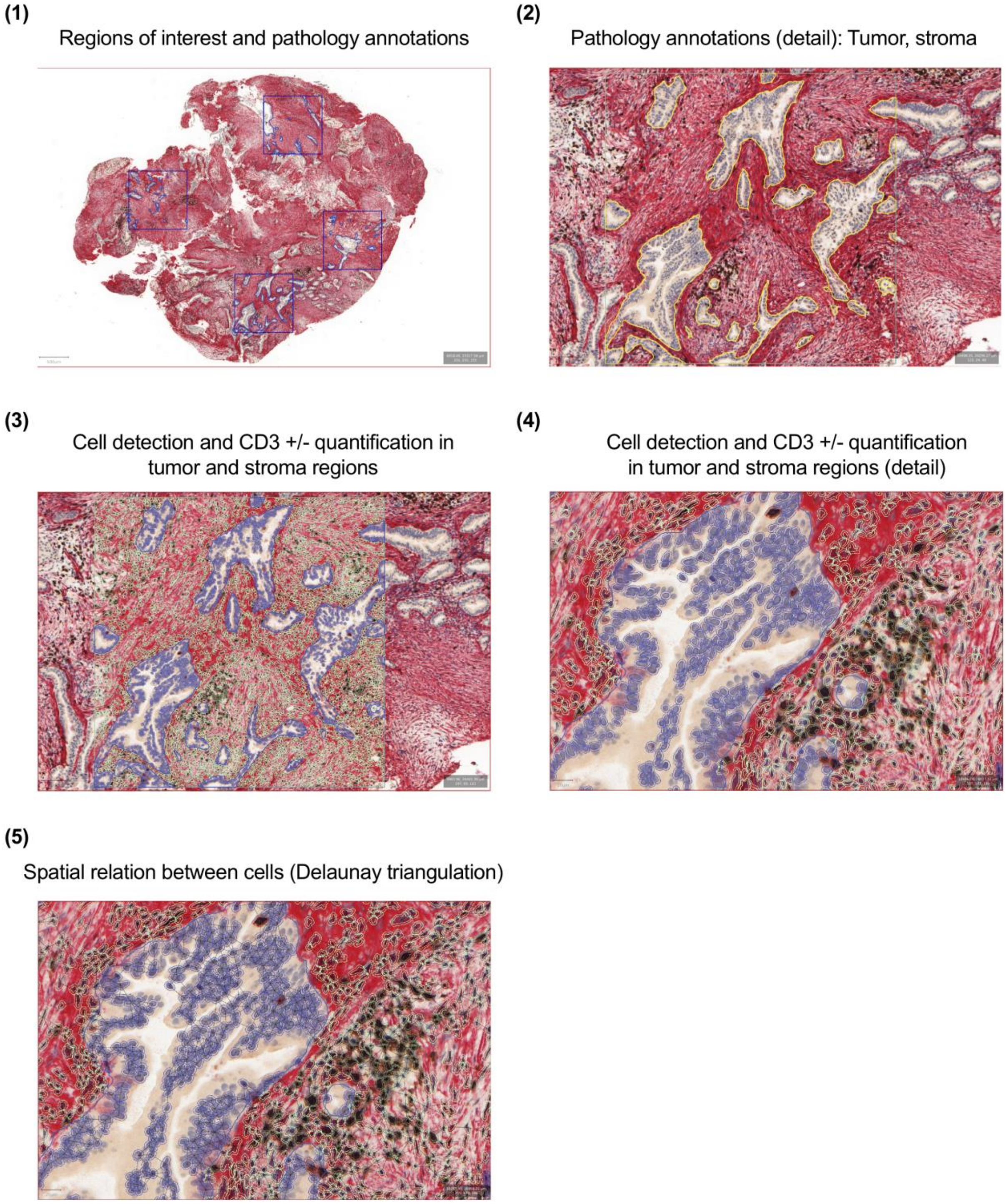

Supplementary Figure S7. Quantitative analysis of the immunohistochemistry stainings using QuPath. Illustrations of the digital image analysis pipeline for quantitation of cells positive for CD3 (1-5) or PD-1 according to DAB staining
